# Mycovirus Hunting Revealed the Presence of Diverse Viruses in a Single Isolate of the Phytopathogenic Fungus *Diplodia seriata* From Pakistan

**DOI:** 10.3389/fcimb.2022.913619

**Published:** 2022-06-29

**Authors:** Haris Ahmed Khan, Paul Telengech, Hideki Kondo, Muhammad Faraz Bhatti, Nobuhiro Suzuki

**Affiliations:** ^1^ Atta-ur-Rahman School of Applied Biosciences (ASAB), National University of Sciences and Technology (NUST), Islamabad, Pakistan; ^2^ Institute of Plant Science and Resources, Okayama University, Kurashiki, Japan

**Keywords:** phytopathogenic fungi, mycovirome, next-generation sequencing, *Diplodia seriata*, Botryosphaeriaceae, ssRNA virus, dsRNA virus, virus/virus interaction

## Abstract

*Diplodia seriata* in the family Botryosphaeriaceae is a cosmopolitan phytopathogenic fungus and is responsible for causing cankers, fruit rot and leaf spots on economically important plants. In this study, we characterized the virome of a single Pakistani strain (L3) of *D. seriata*. Several viral-like contig sequences were obtained *via* a previously conducted next-generation sequencing analysis. Multiple infection of the L3 strain by eight RNA mycoviruses was confirmed through RT-PCR using total RNA samples extracted from this strain; the entire genomes were determined *via* Sanger sequencing of RT-PCR and RACE clones. A BLAST search and phylogenetic analyses indicated that these eight mycoviruses belong to seven different viral families. Four identified mycoviruses belong to double-stranded RNA viral families, including *Polymycoviridae*, *Chrysoviridae*, *Totiviridae* and *Partitiviridae*, and the remaining four identified mycoviruses belong to single-stranded RNA viral families, i.e., *Botourmiaviridae*, and two previously proposed families “*Ambiguiviridae*” and “*Splipalmiviridae*”. Of the eight, five mycoviruses appear to represent new virus species. A morphological comparison of L3 and partially cured strain L3ht1 suggested that one or more of the three viruses belonging to *Polymycoviridae*, “*Splipalmiviridae*” and “*Ambiguiviridae*” are involved in the irregular colony phenotype of L3. To our knowledge, this is the first report of diverse virome characterization from *D. seriata*.

## Introduction

Mycoviruses (fungal viruses) are omnipresent in almost all major fungal and fungal-like organism groups (Herrero et al., 2012; Xie and Jiang, 2014; Garcia-Pedrajas et al., 2019; Nerva et al., 2019a; Sutela et al., 2019; Botella et al., 2020; Myers et al., 2020). Recent mycovirus studies have contributed to a better understanding of virus diversity and evolution (Nerva et al., 2019b; Chiapello et al., 2020; Sutela et al., 2020; Chiba et al., 2021a; Jia et al., 2021; Mu et al., 2021). This can be seen by the recent erection of virus families and even a virus order, such as *Yadokarivirales*, *Hadakaviridae*, *Polymycoviridae Botourmiaviridae* and *Fusariviridae.* Mycoviruses have been classified into 23 families and one genus (*Botybirnavirus*) recognized by the International Virus Taxonomy Committee (ICTV) (Suzuki, 2021). The members of these 24 groups have diverse genome structures with ssDNA (1), reverse-transcribing DNA (retrotransposons) (2), double-stranded (ds) RNA (10), positive-sense (+) single-stranded (ss) RNA (9) and negative-sense (-) ssRNA (2) as their genomic entities (Suzuki, 2021). In addition, there are a myriad of unclassified fungal viruses with peculiar genome organizations. While the fungal virome (mycovirome) is dominated by positive-sense (+)ssRNA viruses and dsRNA viruses, there are no reports of dsDNA mycoviruses (Kondo et al., 2022). Interestingly, many (+)ssRNA mycoviruses and some dsRNA mycoviruses do not form typical rigid virus particles and show different types of capsidless nature (Hillman and Cai, 2013; Kanhayuwa et al., 2015; Zhang et al., 2016; Hisano et al., 2018; Suzuki et al., 2018; Sato et al., 2020a; Sato et al., 2020b).

The majority of mycoviruses lead to asymptomatic infections in their hosts (Ghabrial et al., 2015). However, some mycoviruses induce hypovirulence in phytopathogenic fungi, as exemplified by Chryphonectria hypovirus 1 (CHV1, a hypovirus), which serves as a biological control agent against the destructive chestnut blight (Rigling and Prospero, 2018). An increasing number of mycoviruses are now known to induce phenotypic alterations such as decrease *in vitro* fungal growth and/or virulence, when their hosts are pathogenic to higher organisms (Ghabrial et al., 2015; Okada et al., 2018; Garcia-Pedrajas et al., 2019). A unique example is an ssDNA virus that has been shown to alter the host fungus *Sclerotinia sclerotiorum* from a parasite to an endophyte (Zhang et al., 2020). Moreover, the enhancement of *in vitro* growth, sporulation and/or virulence by mycoviruses has been reported (Kotta-Loizou and Coutts, 2017; Okada et al., 2018; Shah et al., 2020). However, investigation of phenotypic effects by mycoviruses on their host fungi is often hampered by coinfections that are common in various fungi (Hillman et al., 2018). Several types of virus/virus interactions, e.g., synergistic, antagonistic, and mutualistic interactions, have been reported in fungal hosts (Eusebio-Cope and Suzuki, 2015; Hillman et al., 2018). Coinfections of single fungal strains by over ten mycoviruses have been reported for several phytopathogenic fungi. Examples include a strain of *Fusarium poae* coinfected with 16 RNA mycoviruses belonging to 11 viral families (Osaki et al., 2016), and a strain of *Kickxella alabastrina* (the subdivision of Kickxellomycotina) co-infected with 11 RNA mycoviruses (Myers et al., 2020). How these co-infecting mycoviruses interplay remains unknown in these cases. We also have screened many different Pakistani fungal strains from different sources for mycovirus hunting and have reported molecular characterization of some of the discovered novel mycoviruses (Khan et al., 2021a; Khan et al., 2022). The tested fungal strains included the SP1 strain of *Fusarium mangiferae* that was found to be co-infected with 11 mycoviruses (Khan et al., 2021b).

The ascomycetous family Botryosphaeriaceae includes many important phyotpathogenic fungi such as members of the genera *Botryosphaeria*, *Neofusicoccum*, and *Diploidia.* Whereas some members of the first two genera have been explored as viral hosts (Wang et al., 2014; Marais et al., 2021), the third one has not. *Diplodia seriata* (anamorph of *Botryosphaeria obtusa*) is a phytopathogenic ascomycete that causes fruits rot, leaf spot, canker, leaf chlorosis and trunk dieback in many woody and herbaceous plants (Phillips et al., 2007; Farr and Rossman, 2022). In the current study, as an extension of our previous studies, we characterized the virome of a Pakistani strain (L3) of *D. seriata*. The fungal strain was shown to be co-infected by a total of eight RNA mycoviruses spanning four dsRNA virus families and two (+)ssRNA virus families with encapsidated and putative capsidless nature, some of which accommodate members with new virus lifestyles. Hyphal tipping resulted in the elimination of three viruses from the original fungal strain and the restoration of the colony growth.

## Materials and Methods

### Isolation of Fungal Strains and Extraction of dsRNA/Total RNA Fractions

**Figure 1:**
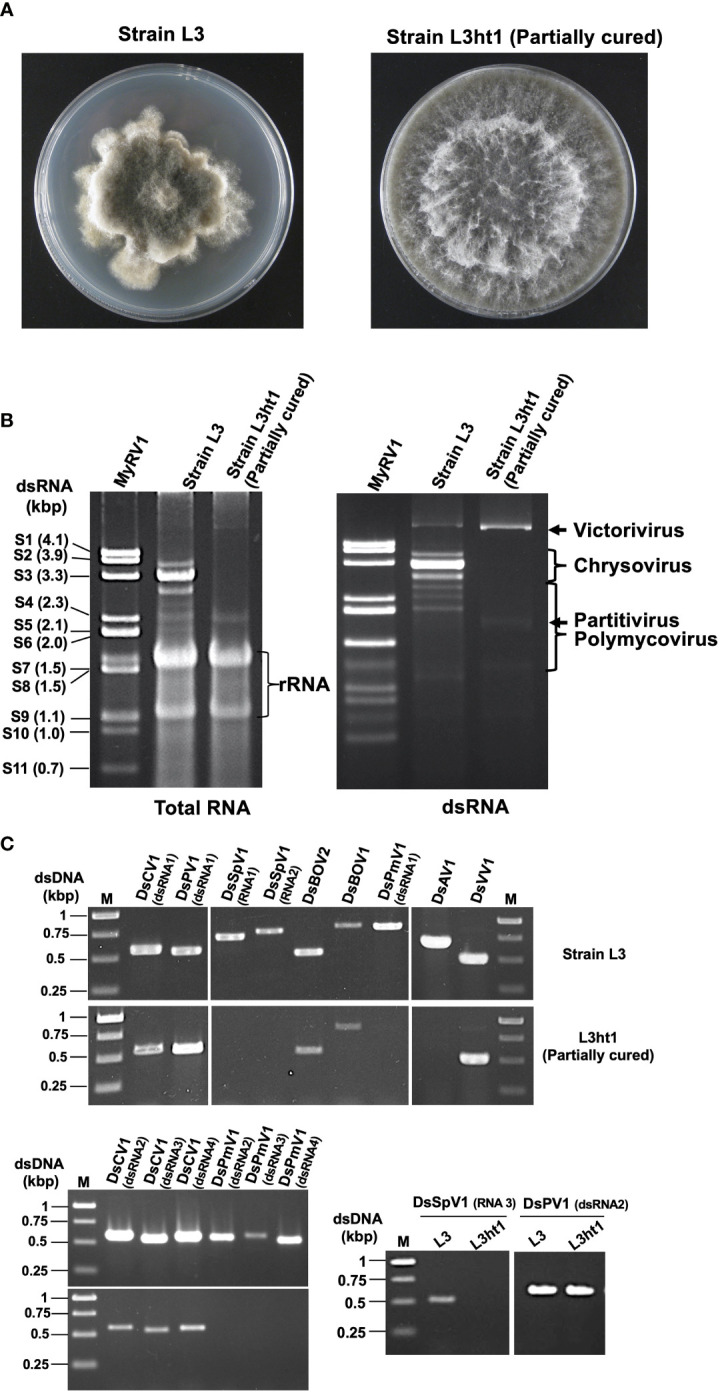
Diverse mycoviruses were detected from *Diplodia seriata* (the strain L3). **(A)** Colony morphology of the strain L3 and L3ht1 grown of PDA media. L3 was partially cured by hyphal tipping to obtain L3ht1. **(B)** Agarose gel electrophoresis of dsRNA and total nucleic acid fractions. DsRNA was purified from an equal amount (5 μg) of total RNA fractions of the two strains and resuspended in an equal amount of distilled water. The genomic dsRNA of MyRV1 (Suzuki et al., 2004) was used as the size standard. **(C)** RT-PCR analysis of the two fungal strains L3 and L3ht1. Total RNA was isolated from the strains and subjected to RT-PCR using primer sets shown in **Supplementary Table S1**. Targeted genomic RNAs are shown on the top of the gel.

The fungal strain L3 (*D. seriata*) was collected and identified during a mycoviral screening survey conducted in 2018 from a diseased leaf of the Lokath plant (Khan et al., 2021b). Some mycoviruses identified as a result of this screening survey have already been published (Khan et al., 2021a; Khan et al., 2021b; Khan et al., 2022). For isolating purified cultures of L3, surface sterilization of leaves was performed with 1% sodium hypochlorite solution and further washing was done using autoclaved sterile distilled water. Samples were air-dried in a safety cabinet and infected parts were inoculated on a fresh potato dextrose agar (PDA, Becton, Dickinson and Co.) plate to obtain fungal cultures. Incubation of fungal cultures was performed at 25°C for 4 to 8 days for growth purposes. Glycerol stocks (40% v/v) of the pure colonies were prepared and stored at -80°C for future use. Fungal identification was performed based on the morphology (**Figure 1**) and internal transcribed spacer (ITS) region sequencing (**Supplementary Figure 1**) (White et al., 1990). Total RNA fractions were obtained as described earlier (Eusebio-Cope and Suzuki, 2015). The classical dsRNA extraction method using cellulose chromatography column was employed to extract dsRNA fractions from the fungal samples (Bhatti et al., 2012; Eusebio-Cope and Suzuki, 2015). The obtained dsRNA fractions were then treated with RQ1 DNase (Promega Corp.) and S1 Nuclease (Thermo Fisher Scientific Inc.) to eliminate genomic DNA and ribosomal RNA contaminations.

### Hyphal Tipping Attempts for Curing Mycoviral Infections

Mycelial plugs taken from one month old mycelia were cultured on 2% water agar plates at 25°C and were allowed to grow under dark conditions. Once growing hyphal tips were confirmed under a dissecting microscope in a clean bench, single hyphal tips from single hyphae were excised with a scalpel and were allowed to grow on new water agar plates. Single sub-isolates were obtained after four rounds of hyphal tipping. All regenerated isolates were cultured on cellophane overlaid PDA to examine for the presence of mycoviruses *via* dsRNA extraction or RT-PCR assay. A total of 30 independent hyphal tip isolates were obtained. Phenotype of the parental strain (L3) and a partially cured hyphal tip subculture (L3ht1) were compared.

### Next-Generation Sequencing of Viral RNA

Total RNA fractions were separately obtained from three dsRNA-positive fungal isolates, L3 (*D. seriata*, a phytopathogen), SP1 (*F. mangiferae*, a phytopathogen) and HK (*Geotrichum candidum*, a soil-inhabitant) and were pooled together as already described by Khan et al. (Khan et al., 2021a; Khan et al., 2021b). Ribosomal RNA depletion, cDNA library construction and RNA sequencing were performed by Macrogen Inc. (Tokyo, Japan) as already described by Khan et al. (Khan et al., 2021b). The assembly of RNA sequencing reads was performed using the Read Mapping algorithm with a default parameter in the CLC Genomics Workbench (version 11, CLC Bio-Qiagen) assembled 42,770 contig sequences were subjected to local BLAST searches to check sequence similarities with deposited virus reference sequences (RefSeq) in the National Center for Biotechnology Information (NCBI) database (data not shown). The presence of mycoviruses corresponding to virus-like contigs was confirmed *via* RT-PCR amplification from total RNA fractions of the L3 strain with virus specific primers (**Supplementary Table S1**). The terminal sequences of viral genomes or segments were achieved through the 3′ RNA ligase mediated rapid amplification of complementary cDNA ends (RLM-RACE) procedure as described by Suzuki et al. (Suzuki et al., 2004). RT-PCR and RACE amplicons were subjected to Sanger sequencing.

### Bioinformatic Analysis

Viral sequence analysis was achieved by performing alignments through GENETYX DNA-processing software. Furthermore, open reading frames (ORFs) were predicted using the online version of the ORF finder program (http://www.ncbi.nlm.nih.gov/gorf/gorf.html). Standard genetic codon usage was selected for the prediction of ORFs for all mycovirus candidates identified in strain L3. Sequence similarity searches were conducted by NCBI BLAST (BLASTn, BLASTx and BLASTp programs) (https://blast.ncbi.nlm.nih.gov/Blast.cgi). NCBI conserved domain database (CDD) (http://www.ncbi.nlm.nih.gov/Structure/cdd/wrpsb.cgi) and motif finder (https://www.genome.jp/tools/motif/) tools were utilized for detecting conserved domains (Marchler-Bauer et al., 2017). Conservation at 5′- and 3′-terminal sequences of the genomes or segments and the motif prediction (I-VIII) for RNA-dependent RNA polymerase (RdRP) core domains were achieved by conducting nucleotide and amino acid sequence alignments, respectively, through the UGENE program (http://ugene.net/) (Okonechnikov et al., 2012). The viral sequences were deposited in the GenBank/ENA/DDBJ database. GC content (%) was calculated through the online server of ENDMEMO program (http://www.endmemo.com/bio/gc.php). The predicted molecular mass of the putative protein encoded by viral genomes or their segments was calculated by an online protein molecular weight estimation program (https://www.bioinformatics.org/sms/prot_mw.html). Transmembrane domains were predicted by TMHMM – 2.0 (https://services.healthtech.dtu.dk/service.php?TMHMM-2.0).

Phylogenetic analyses were performed using RdRP deduced amino acid sequences. Multiple sequence alignments were constructed using the online server of MAFFT (version 7) (https://mafft.cbrc.jp/alignment/server/) (Katoh and Toh, 2008). Unreliable regions of alignments were trimmed using Gblocks ver. 0.91b (Talavera and Castresana, 2007). Maximum likelihood (ML) trees were constructed using the online version (3.0) of PhyML with automatic model selection by SMS (Smart Model Selection) (Lefort et al., 2017) (http://www.atgc-montpellier.fr/phyml-sms/). Bootstrap values with 1000 replicates were used for the analysis and values less than 500 were masked. Trees were rooted at the mid-point for defining the out-group. Final ML trees were visualized using the iTOLL; Interactive Tree Of Life online server (https://itol.embl.de/upload.cgi).

## Results and Discussion

The current study describes the molecular characterization of diverse mycoviruses from a single isolate of *D. seriata* (strain L3) (**Figure 1** and **Supplementary Figure S1**), which was identified as dsRNA positive in the previous screening of Pakistani fungal collections (Khan et al., 2021b). The agarose gel profile of dsRNA extracted from L3 showed the presence of multiple segments with sizes varying from 1.2 to 6.0 kbp (**Figure 1**). We first used the RNA sequencing approach to obtain genomic sequences of mycoviruses harbored by strain L3. NGS sequencing provided many virus-like sequence contigs. Because the NGS data were from three fungal strains (see Materials and Methods), sequence contigs associated with previously reported mycoviruses derived from two other fungi (Khan et al., 2021a; Khan et al., 2021b) were eliminated from the current analyses. RT-PCR analyses assigned a total of 17 virus-like sequence contigs to strain L3. Their RT-PCR pattern is shown in **Figure 1**. Nucleotide sequence and molecular phylogenetic analyses confirmed the presence of eight putative novel mycoviruses belonging to seven viral families (four dsRNA and four (+) ssRNA viruses, respectively) (see below). Identified mycoviruses belonged to the families *Chrysoviridae*, *Polymycoviridae*, *Totiviridae*, *Partitiviridae*, *Botourmiaviridae*, as well as proposed families “*Ambiguiviridae*” and “*Splipalmiviridae*” (**Table 1**). The complete genome sequences were determined for almost all these viruses, while a few genomic segments have yet to be completed and are in the coding-complete status. The accession numbers of the newly determined sequences are presented in **Table 1**.

**Table 1:** Molecular features and BLASTp search results of viruses identified from isolate L3.

Virus/segment (length) (Abbreviation)		Contig No (Read No.)	Accession no.	Hit with highest score in Blastp (accession No.)	Score (bits)	Cover (%)	E-value	Identity (%)
**Diplodia seriata chrysovirus 1 (DsCV1)**								
	RNA1 (3594)	33 (90982)	OM837790	Macrophomina phaseolina chrysovirus 1 (RdRP) (YP_009667008.1)	1516	99%	0.0	63.38%
	RNA2 (3265)	70 (43360)	OM837791	Macrophomina phaseolina chrysovirus 1 (CP) (YP_009667010.1)	793	98%	0.0	43.04%
	RNA3* (3058)	348 (13379)	OM837792	Macrophomina phaseolina chrysovirus 1 (P3) (YP_009667009.1)	459	97%	7e-^144^	33.41%
	RNA4 (3202)	158 (26368)	OM837793	Macrophomina phaseolina chrysovirus 1 (P4) (YP_009667011.1)	1085	99%	0.0	60.57%
**Diplodia seriata polymycovirus 1 (DsPmV1)**								
	RNA1 (2439)	980 (4835)	OM837794	Botryosphaeria dothidea virus 1 (RdRP) (YP_009342446.1)	1124	98%	0.0	75.79%
	RNA2 (2189)	965 (3900)	OM837795	Botryosphaeria dothidea virus 1 (hypothetical) (YP_009342447.1)	1132	100%	0.0	81.56%
	RNA3 (2014)	245 (7883)	OM837796	Botryosphaeria dothidea virus 1 (MTR) (ALZ41796.1)	1069	100%	0.0	83.52%
	RNA4 (1131)	706 (2951)	OM837797	Botryosphaeria dothidea virus 1 (hypothetical) (YP_009342471.1)	419	99%	1e-^145^	77.01%
**Diplodia seriata partititvirus 1 (DsPV1)**								
	RNA1 (1851)	73 (6016)	OM837798	Colletotrichum eremochloae partitivirus 1 (RdRP) (AZT88590.1)	824	100%	0.0	66.67%
	RNA2* (1715)	255 (10271)	OM837799	Penicillium aurantiogriseum partiti-like virus 1 (CP) (ASY04022.1)	460	99%	1e-^154^	50.69%
**Diplodia seriata victorivirus 1 (DsVV1) (4989)**								
** **		995 (2326)	OM837800	Sphaeropsis sapinea RNA virus 2 (CP) (NP_047559.1)	1344	100%	0.0	81.60%
				Sphaeropsis sapinea RNA virus 2 (RdRP) (NP_047560.1)	1138	97%	0.0	85.17%
**Diplodia seriata slipalmivirus 1 (DsSpV1)**								
	RNA1 (2127)	218 (14498)	OM837803	Erysiphe necator associated narnavirus 11 (RdRP) (QJT93743.1)	697	92%	0.0	58.33%
	RNA 2 (2171)	310 (11722)	OM837804	Cryphonectria naterciae splipalmivirus 1 (RdRP) (BCX55510.1)	520	93%	7e-^173^	45.25%
	RNA 3* (900)	175 (16391)	OM837805	Cryphonectria naterciae splipalmivirus 1 (hypothetical protein) (BCX55511.1)	102	81%	7e-^24^	41.13%
Diplodia seriata botourmiavirus 1 (DsBOV1)* (2312)								
		217 (52693)	OM837801	Sclerotinia sclerotiorum ourmia-like virus 16 (RdRP) (QUE49127.1)	476	93%	3e-^157^	45.32%
**Diplodia seriata botourmiavirus 1 (DsBOV2) (2844)**								
		38 (262408)	OM837802	Neofusicoccum parvum ourmia-like virus 1 (RdRP) (QDB74998.1)	1358	100%	0.0	97.19%
**Diplodia seriata ambiguivirus 1 (DsAV1) (3949)**								
		24 (511284)	OM837806	Periconia macrospinosa ambiguivirus 1 (hypothetical) (AZT88665.1)	161	71%	3e-^42^	52.24%
				Erysiphe necator associated ambiguivirus 1 (RdRP) (QKN22641.1)	997	100%	0.0	97.81%

*Coding-complete with terminal sequences undetermined.

### A Novel Chrysovirid

**Figure 2:**
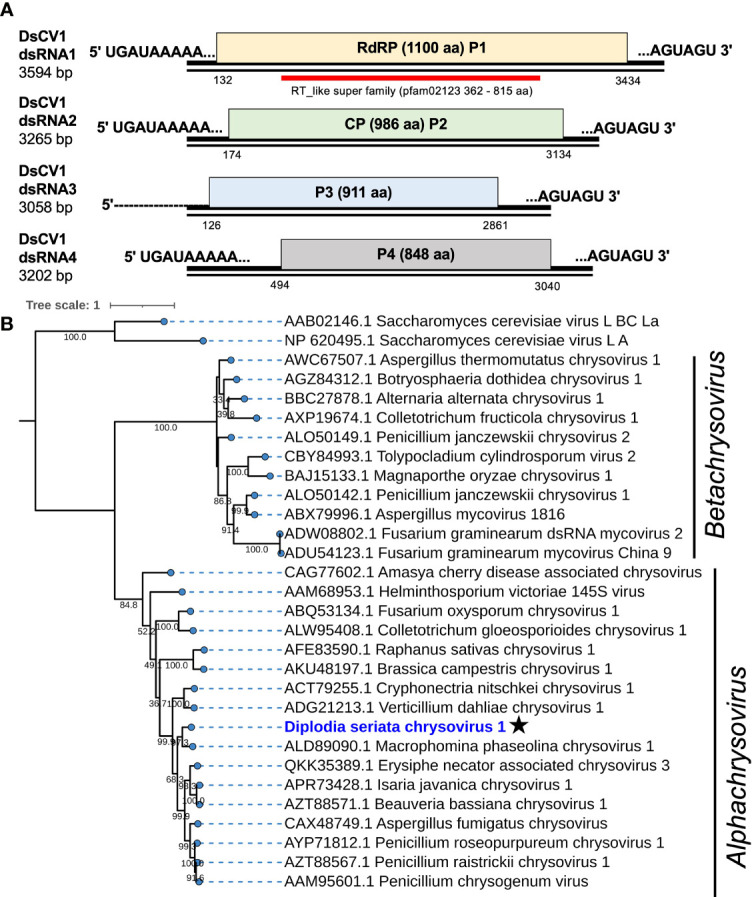
Genome organization and phylogeny of Diplodia seriata chrysovirus 1 (DsCV1). **(A)** Schematic representation of the DsCV1 (an alphachrysovirus) genome identified in *Diplodia seriata* isolate L3. Four DsCV1 genomic segments (dsRNA1 to dsRNA4) encode RNA-directed RNA polymerase (RdRP; P1), capsid protein (CP; P2) and hypothetical proteins encoded by dsRNA3 and dsRNA4. The open reading frames are shown by boxes of different colors in this and subsequent figures. The RdRP domain (RT like super family, pfam00680) was detected in P1 (RdRP) and is represented by a red bar in this and subsequent figures. The complete sequences of all the dsRNA segments were determined, except for dsRNA3, whose 5’-terminal end remained undetermined. **(B)** Phylogenetic tree utilizing the RdRP amino acid sequences derived from DsCV1 and other selected alpha- and betachrysoviruses. Maximum likelihood trees were constructed using PhYML online version 3.0 with 1000 bootstrap replicates under the best-fit model (LG+G+I+F) in this and the subsequent figures, unless otherwise mentioned. Values lower than 500 were masked.

Four contig sequences (contig numbers 33, 70, 348, and 151) (**Table 1**) appeared to represent the four dsRNA segments of a single chrysovirus which we tentatively named Diplodia seriata chrysovirus 1 (DsCV1) (**Figure 2**). DsCV1 dsRNA1 to 4 were numbered with their decreasing sizes (3594 bp, 3265 bp, 3058 bp and 3202 bp, respectively) (**Figure 2**) (**Table 1**). Multiple alignments of terminal nucleotide sequences showed conservation at both termini (except for the dsRNA3 5’ terminal), as reported for other known chrysoviruses (**Supplementary Figure S2A**). CAA rich repeats were also observed at 5′ termini of all dsRNAs, which is commonly observed in chrysoviruses (**Supplementary Figure S2A**). CAA repeat sequences are considered to be translation enhancer elements in tobamoviruses (Jiang and Ghabrial, 2004; Kotta-Loizou et al., 2020).

DsCV1 dsRNA1 encodes for an RdRP (designated P1) of 1100 amino acids (aa) with a predicted molecular weight of 128 kDa. DsCV1 P1 was found to have an RdRP domain (pfam02123, E-value 2.7e-56) predicted from location 362 aa to 815 aa in NCBI CDD search (**Figure 2**) and eight conserved motifs (I–VIII) observed in RdRPs of typical dsRNA viruses (**Supplementary Figure S2B**). A BLASTp search showed that DsCV1 RdRP exhibited a high sequence identity to Macrophomina phaseolina chrysovirus 1 (MpCV1) RdRP (63.4% identity; **Table 1**) (Marzano et al., 2016). Similarly, the other proteins P3 and P4 encoded by DsCV1 dsRNA3 and dsRNA4, respectively, showed the highest aa sequence identity to the corresponding proteins of MpCV1: 33.4% identity for P3 (911 aa, 101 kDa) and 60.6% identity (848 aa, 95.2 kDa) (**Table 1**) (Marzano et al., 2016). P3 and P4 encode hypothetical proteins with unknown functions. The presence of small ORFs upstream of the major ORFs was observed in each genomic segment (except for dsRNA1) of an alphachrysovirus, Cryphonectria nitschkei chrysovirus 1 (CnCV1) (Shahi et al., 2021). No such small ORF was detected in DsCV1 genome segments.

Phylogenetic analysis, based on the complete aa sequence of the RdRP of DsCV1 and selected members of the C*hrysoviridae* family indicated that DsCV1 was phylogenetically close to MpCV1 and clustered with alphachrysoviruses (**Figure 2**). These results are in accordance with the BLASTp search result (**Table 1**). According to ICTV species demarcation criteria for chrysoviruses (≤70 % and ≤53 % identity in RdRP and CP) (Kotta-Loizou et al., 2020), DsCV1 appears to represent a new species of the genus *Alphachrysovirus*, the first chrysovirus infecting the phytopathogenic fungus *D. seriata*. Since CnCV1 is likely to have a very narrow host range (Shahi et al., 2021), it is worth interesting whether DsCV1 and other chrysoviruses have narrow or broad host ranges.

### A Novel Polymycovirid

**Figure 3:**
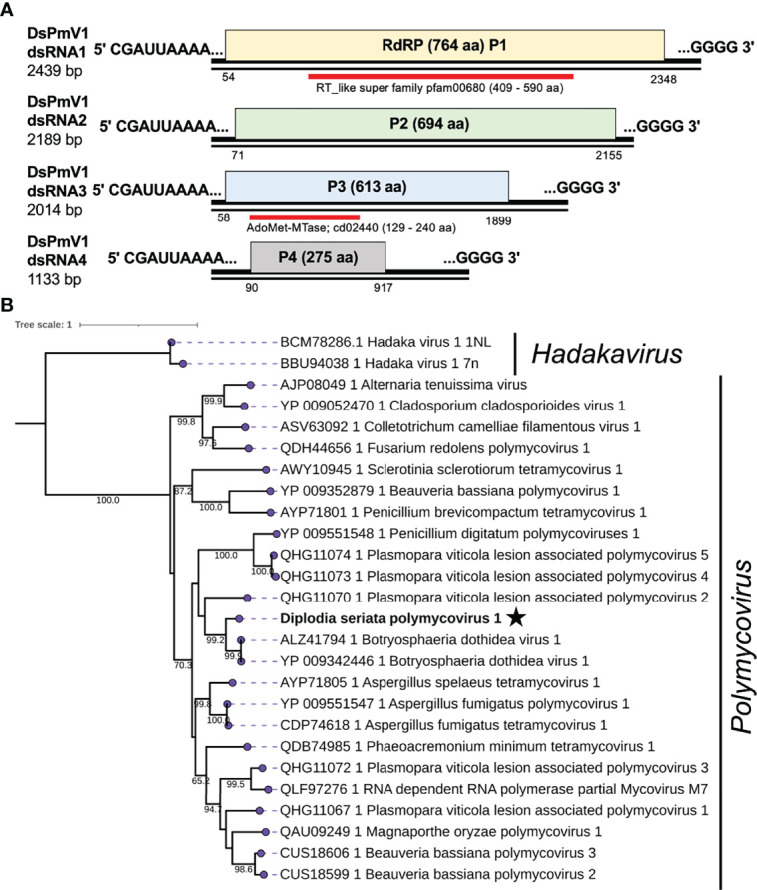
Diagrammatic genome organization and phylogenetic position of Diplodia seriata polymycovirus 1 (DsPmV1). **(A)**. Schematic representation of the DsPmV1genome. The genomic segments RNA1 to dsRNA4 encode RNA dependent RNA polymerase (RdRP; P1), a hypothetical protein P2, methyltransferase (MTR; P3) and a hypothetical protein (P4). P3 was predicted to possess a conserved domain of SAM or AdoMet-MTase (cd02440) and is denoted by the blue bar. The dsRNA4 harbored PASrp region, which is commonly observed in polymycoviruses and is believed to be involved in encapsidation of their genome. Two black lines around the complete genome (pink region) represent the two segments showing the dsRNA nature of the genome. **(B)**. Phylogenetic tree utilizing the RdRP amino acid sequences derived from DsPmV1 and other polymycovirses. Hadaka viruses 1 (HadV1-1NL and 7n) were used as an out-group.

The four contig sequences (contig numbers 980, 965, 245, and 706) were assumed to represent the four dsRNA segments (dsRNA1 to dsRNA4) of a polymycovirus designated as Diplodia seriata polymycovirus 1 (DsPmV1) (**Table 1** and **Figure 3**). DsPmV1 dsRNA 1-4 were 2439 bp, 2189 bp, 2014 bp, and 1133 bp in length, respectively (**Figure 3**), with their predicted GC content ranging of 61–63%. The multiple nucleotide sequence alignment of all four DsPmV1 dsRNAs showed a high degree of conservation at the 5’ and 3’ termini (**Supplementary Figure S3A**). The 5’ termini of all dsRNAs shared 5’-CGAUUAAAACUU…-3’ sequence and the 3’ termini shared conserved 5’-GGGG…-3’ tetranucleotide. DsPmV1 dsRNA1 has a single ORF (designated P1) encoding an RdRP of 764 aa with a predicted molecular weight of 83.6 kDa (**Figure 3**). A motif search of the NCBI CDD revealed P1 has an RdRP domain (fam00680, E-value 7.4e-^08^) spanning amino acid positions 409–590 aa (Koonin and Dolja, 1993) (**Figure 3**) along with eight conserved motifs (I-VIII) of typical RdRPs (**Supplementary Figure S3B**). A conserved GDNQ motif was observed in DsPmV1 P1, which is a peculiar feature of members of the family *Polymycoviridae*. This tetrad is commonly observed in (-)ssRNA viruses in the order *Mononegavirales* (**Supplementary Figure S3B**) (Kanhayuwa et al., 2015; Zhai et al., 2016; Kotta-Loizou and Coutts, 2017; Sato et al., 2020a). A BLASTp search of the NCBI protein database found that DsPmV1 P1 shares 75.8% aa sequence identity with the RdRP of Botryosphaeria dothidea RNA virus 1 (BdRV1) (Hu and Kurgan, 2019; Mahillon et al., 2019) (**Table 1**). DsPmV1 dsRNA2 encodes for the 694 aa protein (designated P2, 74.7 kDa). P2 shows 81.6% aa sequence identity to BdRV1 P2 (**Table 1**), but no conserved motifs were observed in this protein. The dsRNA3-encoded protein (613 aa, designated as P3) has a predicted molecular mass of 66.4 kDa (**Figure 3**) and a motif suggesting putative class I S-adenosylmethionine-dependent methyltransferase (AdoMet-MTase; cd02440) as in the case for other polymycovirus P3 proteins (Kanhayuwa et al., 2015; Zhai et al., 2016; Jia et al., 2017; Sato et al., 2020a). DsPmV1 P3 shares 83.5% aa sequence identity with BdRV1 P3 (**Table 1**). Although the experimental data are not available, the presence of this domain suggests that P3 is involved in capping genomic dsRNA or messenger RNAs as proposed earlier (Kanhayuwa et al., 2015). DsPmV1 dsRNA4 encodes a protein of 275 aa (designated P4, 28.6 kDa) (**Figure 3**). This segment was predicted to contain a proline-alanine-serine rich protein (PASrp) [P(21), A(41), and S(21) residues] and showed 77.0% similarity with P4 of BdRV1 (**Table 1**).

Phylogenetic analysis, based on the complete aa sequence of the RdRPs, suggested that DsPmV1 shows close phylogenetic affinity to BdRV1 in the family *Polymycoviridae* (**Figure 3**). According to polymycovirus species demarcation criteria (≤70 % aa sequence identity in the RdRP) (https://talk.ictvonline.org/files/ictv_official_taxonomy_updates_since_the_8th_report/m/fungal-official/9407), DsPmV1 should be classified as the same species as BdRV1 based on the high level of aa sequence identity. It will be an interesting question whether these two viruses might have co-evolved with their host fungi or whether they might have been horizontally transferred between the two phylogenetically closely related fungi.

### A Novel Partitivirid

**Figure 4:**
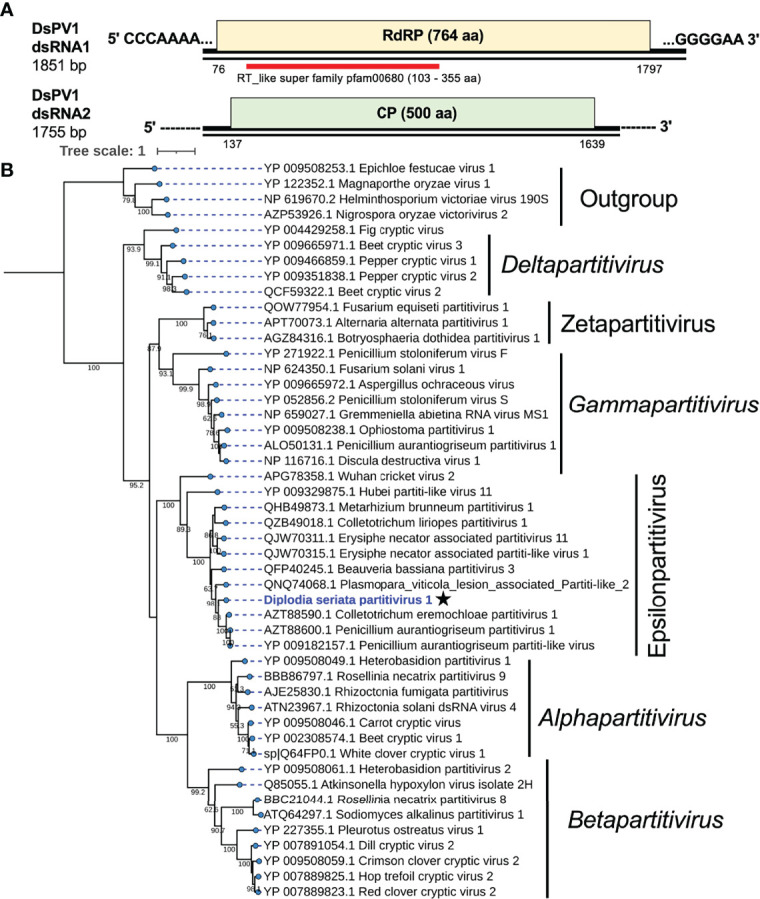
Genome structure and phylogenetic position of Diplodia seriata partitivirus 1 (DsPV1). **(A)** Schematic genome representation of DsPV1 (an epsilonpartitivirus). While the dsRNA1 sequence was fully determined, both termini of dsRNA2 have yet to be determined. **(B)** Phylogenetic tree with the RdRP amino acid sequences derived from DsPV1 and other different groups of partitiviruses. The best fit model (VT+G+I+F) was selected as a substitution model for evaluating the phylogenetic relationships.

Two contig sequences (contig numbers 73 and 225) showed similarities with the dsRNA segments encoding RdRP and CP of already reported partitiviruses, respectively (**Table 1**), and corresponded to the genomic segments of a partitivirus termed Diplodia seriata partitivirus 1 (DsPV1). The genome organization of DsPV1 is shown in **Figure 4**. No interrupted poly(A) stretches or poly(A) tail was observed at the 3′ terminal of DsPV1 segments (**Supplementary Figure S4A**), which is commonly observed at the 3′ terminus of many partitiviruses (Vainio et al., 2018). DsPV1 dsRNAs have 76 nt and 137 nt at the 5′ terminus and 97 nt and 116 nt at the 3′ terminus, which constitute the untranslated regions (UTRs) of dsRNA1 and dsRNA2, respectively (**Figure 4**). DsPV1 shared the conserved terminal sequences, i.e., 5′-CCCAA…-3′ at the 5′ terminus and 5′-…CCCCUUCGGG-3′ at the 3′ terminus with Metarhizium brunneum partitivirus 1 (MbPV1) (**Supplementary Figure S4A**) (accession no. QHB49873) (Wang et al., 2020) (**Table 1**). DsPV1 dsRNA1 encodes RdRP (573 aa), while DsPV1 dsRNA2 encodes CP (**Figure 4**). DsPV1 dsRNA1 (1851 bp) encodes RdRP (573 aa, 66.0 kDa) (**Figure 4**). BLASTp search showed that DsPV1 RdRP exhibits high aa sequence identity to that of Colletotrichum eremochloae partitivirus 1 (CePV1) (66.8%) and to MbPV1 (63.5%) (**Table 1**). We identified six conserved motifs (III-VIII) in the DsPV1 RdRP (**Supplementary Figure S4B**). DsPV1 dsRNA2 comprises of 1755 nts and was predicted to encode for CP. DsPV1 CP showed 50.7% aa sequence identity with Penicillium aurantiogriseum partitivirus 1 (PaPV1).

An RdRP-based phylogenetic tree showed that DsPV1 belongs to the newly proposed genus “*Epsilonpartitivirus*” in the family *Partitiviridae* together with MbPV1, CePV1, PaPV1 and many others (Nerva et al., 2017) (**Figure 4**). The ICTV-set species threshold for partitiviruses is ≤ 90% aa sequence identity in RdRP and ≤ 80% aa sequence identity in CP (Vainio et al., 2018). Based on this criterion, DsPV1 is considered to represent a new species in this proposed genus.

### A Novel Totivirid

**Figure 5:**
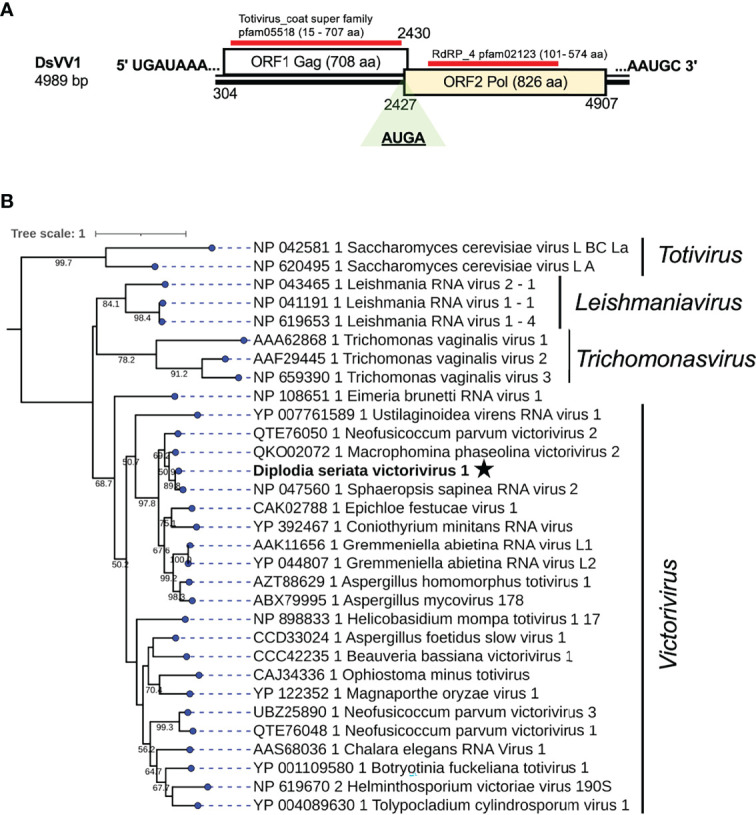
Genome organization and phylogenetic placement of Diplodia seriata victorivirus 1 (DsVV1). **(A)** Schematic representation of the DsVV1genome. The genome of DsVV1 has two large ORFs encoding capsid protein (shown by the blue bar) (coat super family, pfam05518) and RdRP (denoted by the red bar) (RdRP_4, pfam02123). DsVV1 RdRP is likely produced *via* the stop/restart mechanism, in which the tetranuclepotide (*AUG*A) serves as a facilitator (see text). **(B)** RdRP-based phylogenetic analysis of DsVV1 and other members of the family *Totiviridae*.

A contig sequence (contig number 995) showed similarities with members of the genus *Victorivirus* under family *Totivirdae* (**Table 1** and **Figure 1**) and corresponded to an undivided dsRNA genome of a victorivirus named Diplodia seriata victorivirus 1 (DsVV1). The complete genome sequence of DsVV1 comprises of 4989 bp in length and possessed two large ORFs (ORF1 and ORF2), which encode for putative CP (708 aa, 74 kDa) and RdRP (826 aa, 91 kDa) (**Figure 5**). DsVV1 CP and RdRP shared 85.2% and 81.6% aa sequence identity with those of Sphaeropsis sapinea RNA virus 2 (SsRV2) from a pine pathogen *Diplodia pinea* (Preisig et al., 1998) (**Table 1**). A multiple nucleotide sequence alignment showed that DsVV1 shared conservation at both termini with reported vivtoriviruses, Magnaporthe oryzae virus 2 (MoV2; accession no. LC573906) and Sphaeropsis sapinea RNA virus 2 (SsRV2; accession no. AF039080) (**Supplementary Figure S5A**). NCBI CDD search showed that ORF1 (CP) and ORF2 (RdRP) were predicted to have a conserved domain of totivirus CP (pfam05518, spanning from 15 aa to 707 aa) and RdRP domains (pfam02123, spanning from 101 to 574 aa), respectively (**Supplementary Figure S5B**). At the junction of ORF1 and ORF2, a conserved tetranucleotide sequence 5′-*AUG* A-3′ (a potential re-initiation start codon is underlined) was observed (**Figure 5**). This tetranucleotide could be involved in translational termination and re-initiation of the two ORFs, which can express two proteins CP and RdRP separately, as for those of Helminthosporium victoriae 190S virus (Hv190SV) and some other victroiviruses (Li et al., 2015). A pseudoknot structure important for stop/re-initiation translation is predicted to form directly upstream of the tetranucleotide sequence 5′-*AUG* A-3′ at a position similar to that in Hv190SV (Li et al., 2015). As a putative stop/restart facilitator, some victoriviruses and hypoviruses have a pentanucleotide sequence at their junction (5’-UA*AUG*-3’) (Guo et al., 2009; Chiba et al., 2013; Jamal et al., 2019).

Phylogenetic analysis, based on the complete aa sequence of the RdRp (ORF2) of DsVV1 and selected members of the *Totiviridae* family indicated that DsVV1 made clade with reported victoriviruses, though with a low supporting value 50.2% for victoriviruses (**Figure 5**). The ICTV species threshold for victoriviruses is set low: <60%. Thus, it is reasonable to classify DsVV1 in the same species as SsRV2, both of which were isolated from *Diplodia* fungi.

### A Novel Splipalmivirus

Two contig sequences (contig numbers 218 and 310) showed resemblance with narna-like viruses [(+)ssRNA genome], particularly members of the newly proposed family “*Splipalmiviridae*” (Sutela et al., 2020; Sato et al., 2022) (**Table 1**). Splipalmiviruses have been coined for viruses with the *spli*t *palm* (italicized letters are used in the term “splipalmivirus”) domains of RdRP that are encoded separately by different genomic segments (Sutela et al., 2020). The obtained viral sequences likely corresponded to the genomic segments of a splipalmivirus tentatively named Diplodia seriata splipalmivirus 1 (DsSpV1). The complete sequences of DsSpV1 RNA1 and RNA2 shared 12 conserved nucleotides (5′-UUUUGCUUGCGA—3′) and four nucleotides (5′—GUUU-3′) at the 5′ and 3′ termini, respectively (**Supplementary Figure S6A**), which suggest that both contigs represent the genome of a single virus. During data analyses, a third contig sequence (contig number 175) has been detected as a candidate of the third RNA segment of DsSpV1(**Table 1**), however their terminal sequences were still not determined.

**Figure 6:**
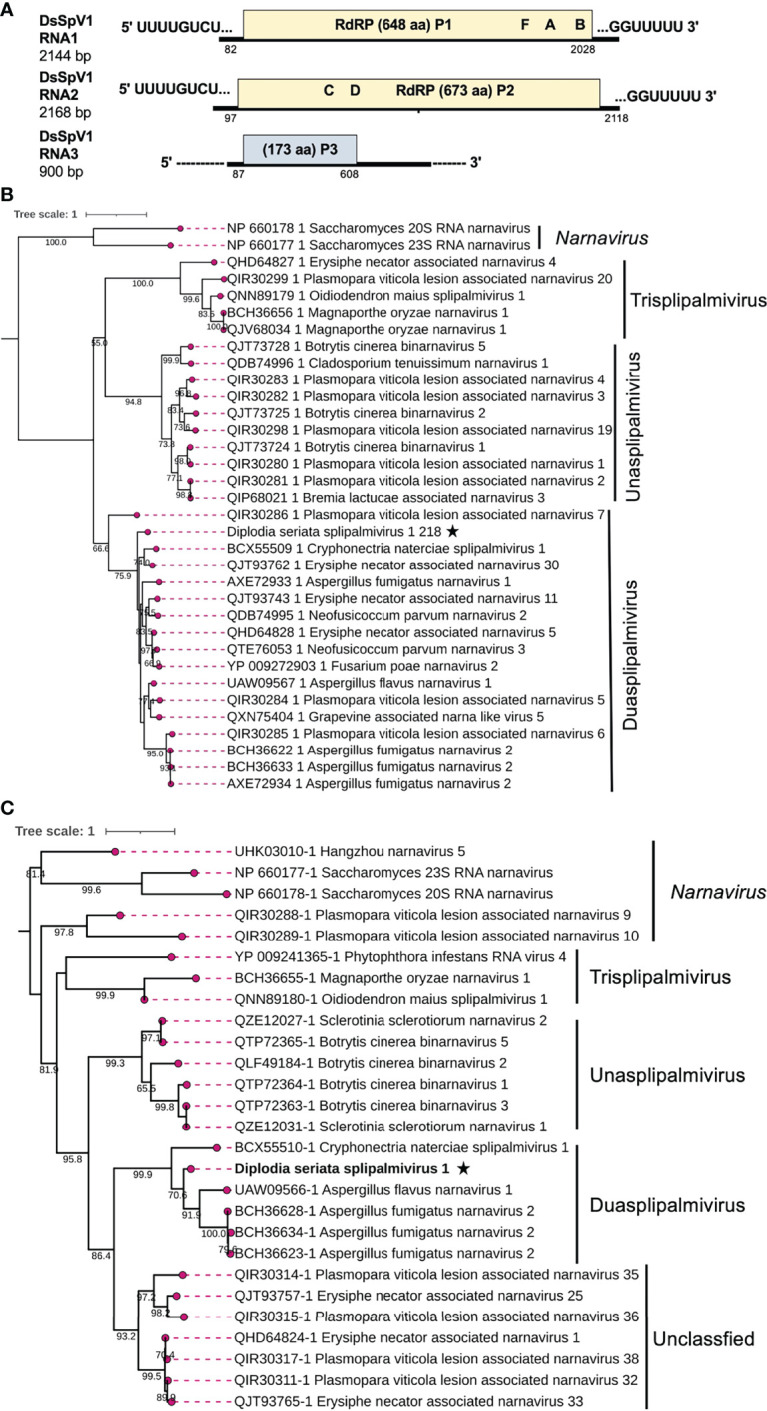
Genome architecture and phylogenetic position of Diplodia seriata splipalmivirus 1 (DsSpV1). **(A)** Schematic representation of the DsSpV1genome. The DsSpV1 genome has three segments encoding for split RdRP (P1 and P2) and a hypothetical protein (P3). While the RNA1 and RNA2 sequences were completely determined, the third segment, RNA3, has yet to be fully determined. **(B, C)** RdRP-based phylogenetic analysis of DsSpV1 and other splipalmiviruses (proposed family “*Splipalmiviridae*”) and narna, and narna-like viruses. The best fit model (LG+G+I+F for P1 and VT+G+F for P2) was selected as a substitution model for evaluating the phylogenetic relationships. The tree was rooted at mid-point, and classical narnaviruses formed an out-group (**B**, **Figure 7**).

DsSpV1 RNA1 (2144 nucleotides, nt) and RNA2 (2168 nt) harbored single ORFs encoding the N-terminal (designated P1, 648 aa and 73.6 kDa) and C-terminal parts (designated P2, 673 aa and 77.2 kDa) of the RdRP, as observed in other splipalmiviruses (**Figure 6**) (Sato et al., 2022). DsSpV1 RNA1-encoded P1 shared aa sequence identity of 58.3% and 56.5% with those of Erysiphe necator associated narnavirus 11 and Cryphonectria naterciae splipalmivirus 1 (CnSpV1), respectively (Ruiz-Padilla et al., 2021; Sato et al., 2022). DsSpV1 RNA2-encoded P2 shared 45.3% and 44.3% aa sequence identity with those of CnSpV1 and Aspergillus flavus narnavirus 1 (AfuNV2), respectively (Zoll et al., 2018; Sato et al., 2022). DsSpV1-P1 contained RdRP motifs G, F, A, and B, while that encoded by DsSpV1-P2 had RdRP motifs C and D (**Supplementary Figure S6B**). The length of RNA1 was smaller than RNA2, as has been observed in the case of CnSpV1 (Sato et al., 2022). The third DsSpV1 RNA (RNA3 candidate) has a size of ~900 nt, which encodes for a hypothetical protein (designated P3, 173 aa and 18.9 kDa). DsSpV1 P3 showed 41.1% aa sequence identity to the CnSpV1 counterpart (P3). Known splipalmiviruses have different numbers of RNA segments ranging from two to seven (Sutela et al., 2020; Chiba et al., 2021a; Jia et al., 2021; Ruiz-Padilla et al., 2021). AfuNV2 and CnSpV1, closely related to DsSpV1, have three and four RNA segments, respectively. We could not find a fourth or additional segment(s) of DsSpV1 in the NGS data set (data not shown).

**Figure 7:**
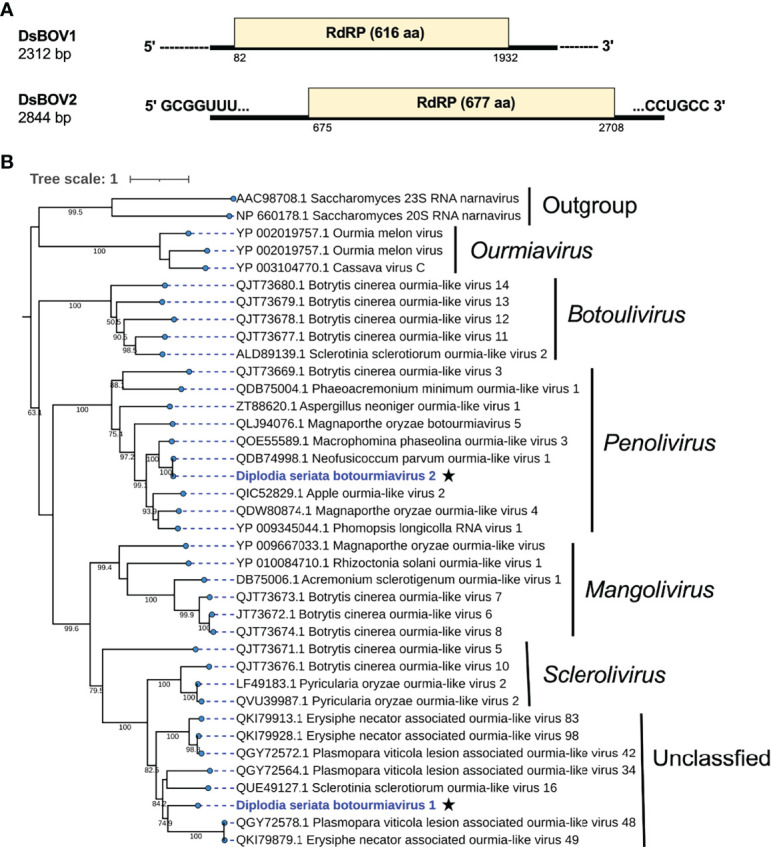
Genome organization and phylogenetic placement of Diplodia seriata botourmiavirus 1 and 2 (DsBOV1 and DsBOV2). **(A)** Schematic genome representation of the DsBOV1 (a sclerolivirus) and DsBOV2 (a penolivirus). Both viruses encode only RdRP. Sequence determination is complete only for DsBOV2. **(B)** Phylogenetic analysis utilizing the RdRP amino acid sequences derived from DsBOV1 and 2 with other members of the family *Botourmiaviridae*. The best fit model (VT+G+I+F) was selected as a substitution model for evaluating the phylogenetic relationships.

To estimate the phylogenetic relationships, the split RdRP sequences encoded by RNA1 and RNA2 of DsSpV1 and other splipalmiviruses, and the representative members of the family *Narnaviridae* were used to construct phylogenetic trees. The results showed that among the three proposed splipalmiviral genera (Sato et al., 2022), DsSpV1 was placed in a clade with high branch support values of 75.9% (for P1) and 99.9% (for P2) with duasplipalmiviruses (**Figure 6C**). Currently, there is no established species demarcation criteria set by ICTV for splipalmiviruses. DsSpV1 is divergent enough from previously reported duasplipalmiviruses (~58.3% or ~45.3% for the split RdRP aa sequence identity) to classify in a new species in the proposed genus “*Duasplipalmivirus*”. DsSpV1 will be among rare splipalmivirids that are characterized biologically (see below); its horizontal transmission was confirmed.

### Novel Botourmiavirids

**Figure 8:**
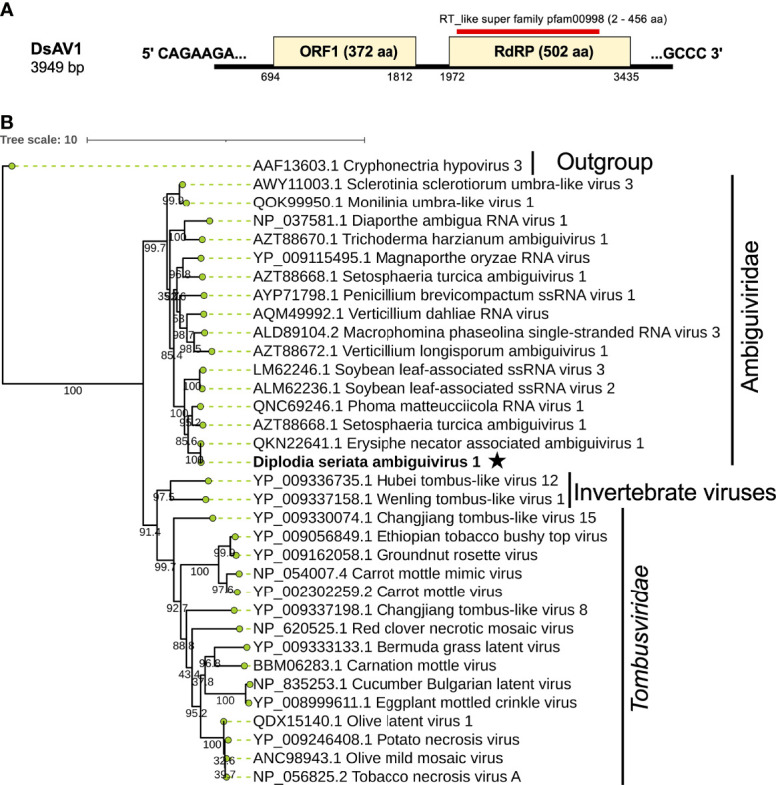
Genome architecture and phylogenetic position of Diplodia seriata ambiguivirus 1 (DsAV1). **(A)** Schematic representation of DsAV1 (an ambiguivirus). The undivided DsAV1 (+)ssRNA genome possesses two ORFs encoding a hypothetical protein (ORF1) and an RdRP (RdRP-ORF2). **(B)** Phylogenetic analysis with the RdRP aa sequences derived from DsAV1 and other members of *Tombusviridae* and the proposed family “*Ambiguiviridae*” (Gilbert et al., 2019).

Two contig sequences (contig numbers 217 and 38) showed similarities with members of family *Botourmiaviridae* (**Table 1**). These two putative mycoviruses were tentatively named as Diplodia seriata botourmiavirus 1 and 2 (DsBOV1 and 2). DsBOV1 and DsBOV2 possess single ORFs that would encode RdRPs of 616 aa (69.7 kDa) and 677 aa (75.7 kDa), respectively (**Figure 8**). DsBOV2 RNA showed conservation at the termini with already reported Neofusicoccum parvum ourmia-like virus 1 (NpOLV1) (accession no. MK584837) (**Supplementary Figure S7A**) (Nerva et al., 2019b). The DsBOV1 RNA encoded-protein showed aa sequence identity of 45.3% to Sclerotinia sclerotiorum ourmia-like virus 16 RdRP (Jia et al., 2021), while DsBOV2 RNA encoded-protein showed 97.2% aa sequence identity to NpOLV1 RdRP (**Table 1**). The DsBOV1 and 2 proteins shared eight conserved motifs of RdRP with other reported botourmiaviruses (**Supplementary Figure S7B**).

Phylogenetically, DsBOV1 made clade with scleroliviruses (genus *Sclerolivirus*) and DsBOV2 made clade with penoliviruses (genus *Penolivirus*) within family *Botourmiaviridae* (**Figure 8**). Based on the ICTV species demarcation criteria (<90% RdRP aa sequence identity) (Ayllon et al., 2020), DsBOV1 should represent a novel species within the genus *Sclerolivirus* in the family *Botourmiaviridae*, while DsBOV2 should be classified as a strain belonging to the same species as NpOLV1 within the genus *Penolivirus*.

### A Novel Ambiguivirus

A contig sequence (contig number 24) showed sequence similarities with members of the proposed “*Ambiguiviridae”* and family *Tombusviridae* (Gilbert et al., 2019). The complete genome of the virus, tentatively named Diplodia seriata ambiguivirus 1 (DsAV1), comprised 3949 nt and harbored two noncontiguous ORFs (ORF1 and ORF2) encoding 372 aa (P1, 41.1 kDa) and 502 aa (P2, 55.7 kDa), which were separated by 160 nt (**Figure 8A**). A database search with BLASTp showed that the putative RdRP aa sequence (ORF2) of DsAV1 shares 97.8% sequence identity to Erysiphe necator associated ambiguivirus 1 (EnAAV1) (accession no. QKN22641, a partial genome sequence with 2856 nt length) (Ruiz-Padilla et al., 2021). DsAV1 ORF1-encoded hypothetical protein shared 51.0% identity to that of Phoma matteucciicola RNA virus 1 (accession no. QNC69246) (Zhou et al., 2021). DsAV1 P2 contained five conserved motifs (IV-VIII) of RdRP domain (pfam00998 spawning from map positions 2 to 456) (**Figure 8A**) (**Supplementary Figure S8A**). An interesting observation was that RdRPs of DsAV1 and other known ambiguiviruses have a GDNA tetrad in motif IV in comparison to the GDD motif found in most (+)ssRNA and dsRNA viruses (REF). Generally, the GDD triplet has a detrimental effect on viability in (+)ssRNA viruses (Vazquez et al., 2000; Das et al., 2021), while dsRNA polymycovirus or ssRNA hadaka virus RdRPs have GDNQ in place of GDD, as mentioned before (Sato et al., 2020a; Sato et al., 2020b Kanhayuwa et al., 2015) (**Supplementary Figure S8A**). A total of four transmembrane domains were observed at the N-terminus of the ORF1 (P1) protein (**Supplementary Figure S8A**), as has been observed in other related viruses (Ai et al., 2016; Canizares et al., 2018). This suggests these domains are important for (+)ssRNA viruses and are reported to play a role in anchoring viral proteins to host membrane, as proposed for Magnaporthe oryzae virus A (Ai et al., 2016) and Verticillium dahliae RNA virus 1 (Canizares et al., 2018).

Phylogenetic analysis with RdRP sequences showed that DsAV1 is grouped together with other reported fungal RNA viruses (ambiguiviruses) and well-separated from other related viruses from plants (family *Tombusviridae*) and insects (unassigned viruses) (**Figure 8B**). Classification of possible members of the recently proposed family “*Ambugiviridae*” has not been established. Based on the high degree of sequence similarity (~60.0% RdRP aa sequence identity), both DsAV1 and EnAAV1 should belong to a same novel species in the proposed family. Biological information is unavailable for all reported ambiguiviruses. In this regard, it is of importance that DsAV1 could be eliminated from and re-introduced into the host fungus (see below).

### Elimination of Some Viruses From *D. seriata* Strain L3

As shown above, *D. seriata* strain L3 was found to harbor at least eight mycoviruses. To check the possible effect of these viruses on the host fungal strain, we attempted to cure it of the viruses by a few methods. While single spore isolation is often used for this purpose, L3 turned out not to produce spores on PDA media. We then performed hyphal tipping and over 30 subcultures were tested for virus infection. One subculture termed L3ht1, showed a colony morphology different from L3 (**Figure 1**), and was predicted to have lost a few mycoviruses originally carried in the L3 strain. This could be seen from a dsRNA profile, showing the absence of expected dsRNAs of DsCV1 (3594 bp, 3265 bp, 3058 bp and 3202 bp) and DsPmV1(2439 bp, 2189 bp, 2014 bp, and 1133 bp) in L3ht1 that were present in L3 (**Figure 1**). Note that some (+)ssRNA viruses, such as splipalmiviruses do not accumulate replicative dsRNA forms in infected fungal host cells (Sato et al., 2022). To confirm which mycoviruses were eliminated in L3ht1, RT-PCR was carried out using the nine primer sets for eight mycoviruses, including split RdRP encoded segments, as listed in **Supplementary Table S1**. Consequently, the L3ht1 was shown to harbor five mycoviruses, DsVV1, DsCV1, DsPV1, DsBOV1 and DsBOV2, and to have lost three other mycoviruses, i.e., DsPmV1, DsSpV1 and DsAV1 (**Figure 1**). For multisegmented RNA viruses such as DsCV1, DsPV1, DsPmV1 and DsSpV1, consistent RT-PCR results were obtained in which different primer sets targeting different genomic segments of a virus of interest were used (**Figure 1**). Gel electrophoretic analyses of dsRNA and total nucleic acids suggested that whereas L3ht1 accumulated DsVV1 dsRNA more than L3; an opposite trend was observed for DsCV1 (**Figure 1**). This interesting phenomenon warrants further investigation but suggests antagonistic and synergistic interactions between the eliminated viruses and the viruses whose accumulation was enhanced and suppressed in L3ht1, respectively.

Although we could not isolate virus-free subcultures, phenotypic comparison of the partially cured strain L3ht1 showed milder symptoms, increased colony diameter and regular colony margin, as compared to the original strain L3 (data not shown). Thus, this suggests that one or more of the three mycoviruses is involved in the symptom induction. However, it remains unknown how the respective eliminated mycoviruses contribute to the phenotypic alterations.

## Conclusion

Thus far, only one mycovirus (Diplodia seriata betaendornavirus 1, DsEV1) has been reported from *D. seriata* (Nerva et al., 2019b). Here we report the characterization of co-infection of a single strain of *D. seriata* from Pakistan by eight mycoviruses. Of the eight identified mycoviruses, DsCV1, DsPV1, DsSpV1, DsBOV1 and DsAV1 appear to represent new mycovirus species based on the species demarcation criteria set by the ICTV or the high degree of their sequence diversity. The remaining three mycoviruses are putative new strains of previously recognized mycoviral species. The identified mycoviruses belong to the families *Chrysoviridae*, *Polymycoviridae*, *Totiviridae*, *Partitiviridae* and *Botourmiaviridae*, and the proposed families “*Ambiguiviridae”* and “*Splipalmiviridae*”. As stated above, fungal virus hunting studies revealed new genome organizations and new virus lifestyles. Particularly, polymycoviruses and splipalmiviruses are considerably peculiar among the viruses discovered in this study. Polymycoviruses are phylogenetically closely related to hadakaviruses and relatively distantly related to caliciviruses with (+)ssRNA genomes (Kanhayuwa et al., 2015; Sato et al., 2020b; Khan et al., 2021a). However, polymycoviruses are classified as dsRNA viruses, because they are likely infectious in the form of dsRNA deproteinized and associated with PASrp as well (Kanhayuwa et al., 2015). It is noteworthy that DsPmV1 belongs to the family *Polymycoviridae*, whose infectivity as dsRNA needs to be verified. The closest relative of DsPmV1 is Botryosphaeria dothidea virus 1 (BdRV1) (Zhai et al., 2016), which has five genomic dsRNA segments. We failed to detect a fifth dsRNA of DsPmV1 by a similarity-based search of our NGS data.

“Splipalmivirus” is a recently proposed group of (+)ssRNA viruses with bifurcate RdRP domains. Phylogenetically, splipalmiviruses belong to the phylum *Lenarviricota*, which has been expanded rapidly, and most closely related to the established family *Narnaviridae*. A number of eukaryotic *Lenarviricota* members including splipalmiviruses, have been exponentially reported from various filamentous fungi, insects and plants, but most of them have yet to be characterized biologically (Sutela et al., 2020; Chiba et al., 2021a; Chiba et al., 2021b; Jia et al., 2021; Ruiz-Padilla et al., 2021; Sato et al., 2022). One of the best-studied fungal viruses is Saccharomyces 23S RNA narnavirus (ScNV23S), which has an undivided (+)ssRNA genome and exists in infected cells as a capsidless RNA/RdRP complex at a 1:1 molecular ratio (Solorzano et al., 2000; Esteban and Fujimura, 2003; Wickner et al., 2013). A variety of genome types are now detectable that include narnaviruses with multi-segmented or ambisense nature (Sutela et al., 2020; Kondo et al., 2022). The most peculiar narnavirus type is splipalmiviruses because no viruses with divided RdRP domains are found in other members of the kingdom *Orthornavirae*. Their RNA segment numbers vary from two to seven. Although three segments of DsSpV1 have been identified in this study, we cannot rule out that the virus has additional unidentified segments. There are several other open questions about splipalmiviruses. For example, where do splipalmiviruses replicate within host cells? Is association of the two splipalmivirus-encoded proteins with the separate RdRP domains necessary for RNA synthesis?

Interesting virus/virus interactions are expected in strain L3 co-infected with eight viruses, because in partially cured strain L3ht1 an enhanced accumulation of DsVV1 was observed (**Figure 1**). An antagonistic interaction appeared to exist between DsVV1 and one or more of the three removed viruses, i.e., DsPmV1, DsSpV1 and DsAV1. One of the best-studied antagonistic virus/virus interactions in fungi is that between Rosellinia necatrix victorivirus 1 (RnVV1, a victorivirus) and Cryphonectria hypovirus 1 Δp69 (CHV1-Δp69, a hypovirus mutant lacking an RNA silencing suppressor) or mycoreovirus 1 (MyRV1, a mycoreovirus) (Chiba and Suzuki, 2015). RnVV1 is eliminated or severely suppressed in replication by the RNA silencing status highly activated by co-infecting CHV1-Δp69 or MyRV1. A synergistic interaction was anticipated between DsCV1 and one or more of the three eliminated viruses, because in L3ht1 DsCV1accumulation was decreased (**Figure 1**). This may be a reminiscent of the synergism between a hypovirus and a mycoreovirus (Sun et al., 2006). One or more of the three eliminated viruses are implicated in the abnormal colony morphology (**Figure 1**). As DsPmV1 and DsSpV1 belong to newly established or proposed virus families, for most members, biological information is unavailable. Therefore, this study provides a platform for further investigations into molecular and biologic virus/virus interactions among the detected viruses.

## Data Availability Statement

The datasets presented in this study can be found in online repositories. The names of the repository/repositories and accession number(s) can be found in the article/**Supplementary Material**.

## Author Contributions

HAK: Sampling, investigation, writing - original draft. PT: Investigation and results interpretation. HK: Investigation, results interpretation, reviewing and editing original draft. MFB and NS: Conceptualization, supervision, results interpretation, writing – reviewing and editing- original draft. All authors contributed to the article and approved the submitted version.

## Funding

This investigation was partly supported by HEC NRPU grant (No-20-4109/R&D/HEC/14/847 awarded to MFB) and NUST students research, Grants-in-Aid for Scientific Research (A) and Grants-in-Aid for Scientific Research on Innovative Areas from the Japanese Ministry of Education, Culture, Sports, Science and Technology (KAKENHI 21H05035, 17H01463, 16H06436, 16H06429 and 16K21723 to NS and HK).

## Conflict of Interest

The authors declare that the research was conducted in the absence of any commercial or financial relationships that could be construed as a potential conflict of interest.

## Publisher’s Note

All claims expressed in this article are solely those of the authors and do not necessarily represent those of their affiliated organizations, or those of the publisher, the editors and the reviewers. Any product that may be evaluated in this article, or claim that may be made by its manufacturer, is not guaranteed or endorsed by the publisher.
