# Supplementary material for: Mycovirus Hunting Revealed the Presence of Diverse Viruses in a Single Isolate of the Phytopathogenic Fungus Diplodia seriata From Pakistan
Source: Front Cell Infect Microbiol. 2022 Jun 29;12:913619. doi: 10.3389/fcimb.2022.913619 (PMC9277117; doi:10.3389/fcimb.2022.913619)
Supplement: Supplementary file 1 [file DataSheet_1.pdf]

Supplementary information for

Mycovirus hunting revealed the presence of diverse viruses in a single isolate of the phytopathogenic fungus *Diplodia seriata* from Pakistan

Haris Ahmed Khan[1], Paul Telengech[2], Hideki Kondo[2], Muhammad Faraz Bhatti[1]*, Nobuhiro Suzuki[2]*

1. Atta-ur-Rahman School of Applied Biosciences (ASAB), National University of Sciences and Technology (NUST), H-12, 44000, Islamabad, Pakistan.
2. Institute of Plant Science and Resources, Okayama University, Kurashiki, 710-0046, Japan.

Running title: **Virome of a Pakistani isolate of *Diplodia seriata***

*Corresponding authors

Nobuhiro Suzuki

Institute of Plant Science and Resources, Okayama University, Kurashiki, 710-0046, Japan.

e-mail: nsuzuki@okayama.u-ac.jp

Muhammad Faraz Bhatti

Atta-ur-Rahman School of Applied Biosciences (ASAB), National University of Sciences and Technology (NUST), H-12, 44000, Islamabad, PAKISTAN.

e-mail: mfbhatti@asab.nust.edu.pk

Supplementary Figures: 8

Supplementary Table : 1

Supplementary Figures

Supplementary Figure 1. Phylogenetic analysis of isolate L3 (*Diplodia seriata*) utilizing its internal transcribed spacer sequence (ITS) and other isolates within the genera *Diplodia* and *Botryosphaeria*. A maximum-likelihood tree was constructed with a best fit model using 1000 bootstrap value replicates. Values less than 500 were masked and the tree was visualized using an online version of iTOLL (ver. 6.0) (see Materials and Methods). The position of L3 is indicated by the red arrow.

Supplementary Figure 2. Molecular features of Diplodia seriata chrysovirus 1 (DsCV1). (A) A sequence comparison of the terminal untranslated regions (UTRs) of all four genomic segments (dsRNA1 to dsRNA4). The 5'-terminal sequence 5'- UGAUAAAAA---3' was conserved among all three completely sequenced segments. The 5' terminal of dsRNA3 could not be determined *via* RACE. The 3'-terminal sequence 5'----CCUUUAGUAGU-3' was found strictly conserved. (B) Conserved motifs in the RdRP domain of DsCV1 and other chrysoviruses. A total of eight conserved motifs (I-VIII) were observed. Asterisks (*) indicate identical amino acid residues, ":" and "." indicate conserved and semi-conserved amino acid residues. The level of conservation is shown above the aligned sequences as rectangles (silver color). The RdRP conserved motifs are shown in the same manner with the same symbols in the subsequent figures. Abbreviations of viruses utilized in this study are as follows: Diplodia seriata chrysovirus 1 (DsCV1; OM837790) Fusarium oxysporum f. sp. dianthi mycovirus 1 (FodV1; YP 009158913.1), Fusarium graminearum mycovirus China 9 (FgV-ch9; ADU54123.1), Fusarium graminearum dsRNA mycovirus 2 (FgV2; ADW08802.1), Botryosphaeria dothidea chrysovirus 1 (BdCV1; ARE59228.1,Tolypocladium cylindrosporum virus 2 (ToCV2; CBY84993.1), Aspergillus mycovirus 1816 (AsV1816; ABX79996.1), Amasya cherry disease associated chrysovirus (ACDCV; CAG77602.1), Fusarium oxysporum chrysovirus 1 (FoCV1; YP 009665200.1), Cryphonectria nitschkei chrysovirus 1 (CnV1; BBJ21307.1), Penicillium chrysogenum virus (PcV; YP 392482.1), Cryphonectria nitschkei chrysovirus 1 (CnV1; BBJ21307.1).

Supplementary Figure 3. Molecular features of Diplodia seriata polymycovirus 1 (DsPmV1). (A) Terminal sequences of the DsPmV1 genomic segments (dsRNA1 to dsRNA4). The 5'-CGAUUAAAACUU---3' was conserved among all segments. The 3' tetra-nucleotide 5'----GGGG-3' sequence was found conserved. (B) Conserved motifs in RdRP domain of DsPmV1 and other polymycoviruses. The viruses included in the analyses are: Diplodia seriata polymycovirus 1 (DsPmV1; OM837794) Plasmopara viticola lesion associated polymycovirus 1 (PvaPmV1; QHG11067.1), Beauveria bassiana polymycovirus 2 (BbPmV2; CUS18599.1), Magnaporthe oryzae polymycovirus 1 (MoPmV1; QAU09249.1), Aspergillus fumigatus polymycovirus 1 (AfuPmV1; YP 009551547.1), Botryosphaeria dothidea virus 1 (BdRV1; YP 009342446.1), Penicillium janthinellum polymycovirus 1 (PjPmV1; BCJ03666.1), Colletotrichum camelliae filamentous virus 1 (CcFV-1; ASV63092.1).

Supplementary Figure 4. Genomic terminal sequences and RdRP motifs of Diplodia seriata partitivirus 1 (DsPV1). (A) Comparison of the terminal sequences of DsPV1 with Metarhizium brunneum partitivirus 1(MbPV1) (accession no. QHB49873.1). The 5' penta- and 3' deca-nucleotides, 5'-CCCAA---3' and 5'---CCCCUUGGGG-3', were conserved between the two partitiviruses. (B) Conserved motifs in the RdRP domain of DsPV1 and other partitiviruses. The viruses included in the analyses are: Diplodia seriata partitivirus 1(DsPV1; OM837798), Metarhizium brunneum partitivirus 1 (MbPV1; QHB49873.1), Beauveria bassiana partitivirus 3 (BbPV3; QFP40245.1), Colletotrichum eremochloae partitivirus 1 (CePV1; AZT88590.1), Rhizoctonia solani partitivirus virus 5 (RsPV5; AZQ25369.1), Beihai barnacle virus 12 (BbV12; YP 009333370.1).

Supplementary Figure 5. Molecular features of Diplodia seriata victorivirus 1 (DsVV1). (A) Alignment of the terminal sequences of DsVV1 with Magnaporthe oryzae virus 2 (MoV2; LC573906.1) and Sphaeropsis sapinea RNA virus 2 (SsRV2; AF039080.1). No conservation was observed at the 5' terminal region among the three closely related viruses, while the tetra-nucleotide, 5'-GAUA---3' is shared by DsVV1 and MoV2. The 3' penta-nucleotide 5'---AAUGC-3' was conserved among the three viruses. (B)

RdRP conserved motifs of DsPV1 and other victoriviruses. The viruses included in the analyses are: Diplodia seriata victorivirus 1 (DsVV1; OM837800) Neofusicoccum parvum victorivirus 1 (NpVV1; QTE76048.1), Rosellinia necatrix victorivirus 1 (RnVV1; YP 008130308.1), Ustilaginoidea virens RNA virus 1 (UvRV1; YP 007761589.1), Macrophomina phaseolina victorivirus 2 (MpVV2; QKO02072.1), Phomopsis longicolla totivirus 1 (PlTV1; ALD89108.1), Alternaria arborescens victorivirus 1 (AbVV1; YP 009553478.1), Coniothyrium minitans RNA virus (CmRV; YP 392467.1), Penicillium digitatum virus 1 (PdV1; AMY26886.1), Magnaporthe oryzae virus 2 (MoV2; BBG92298.1), Sphaeropsis sapinea RNA virus 2 (SsRV2; NP 047560.1), Macrophomina phaseolina victorivirus 2 (MpVV2; QKI37143.1), Neofusicoccum parvum victorivirus 2 (NpVV2; QTE76050.1).

Supplementary Figure 6. Genomic terminal sequences and RdRP motifs of Diplodia seriata splipalmiviruses 1 (DsSpV1). (A) A comparison of the untranslated regions (UTRs) of the DsSpV1. The genomic segments RNA1 and RNA2 have relatively well-conserved nucleotides at both termini. DsSpV1 RNA3 remained undetermined. (B) Conserved motifs in RdRP domain of DsSpV1, other splipalmiviruses and narna-like viruses. The viruses included in the analyses of motifs G, F, A and B are: Diplodia seriata splipalmivirus 1 (DsSpV1; OM837803) Erysiphe necator associated narnavirus 4 (EnNV4; QJT93743.1), Aspergillus fumigatus narnavirus 1 (AfuNV1; AXE72933.1), Neofusicoccum parvum narnavirus 2 (NpNV2; QDB74995.1), Aspergillus flavus narnavirus 1 (AfNV1; UAW09567.1), Cryphonectria naterciae splipalmivirus 1 (CnSpV1; BCX55509.1), Magnaporthe oryzae narnavirus 1 (MoNV1; QJV68034.1), Oidiodendron maius splipalmivirus 1 (OmSpV1; QNN89179.1), Plasmopara viticola lesion associated narnavirus 20 (PvlaNV20; QIR30299.1), Neofusicoccum parvum narnavirus 3 (NpNV3; QTE76053.1), Aspergillus fumigatus narnavirus 2 (AfuNV2; BCH36622.1). The viruses included in the analyses of motifs C and D are: Diplodia seriata splipalmivirus 1 (DsSpV1; OM837804) Cryphonectria naterciae splipalmivirus 1 (CnSpV1; BCX55510.1), Magnaporthe oryzae narnavirus 1 (MoNV1; BCH36655.1), Oidiodendron maius splipalmivirus 1 (OmSpV1; QNN89180.1), Botrytis cinerea binarnavirus 2 (BcBNV2; QLF49184.1), Aspergillus fumigatus narnavirus 2a (AfuNV2a; BCH36634.1), Aspergillus fumigatus narnavirus 2b (AfuNV2b; BCH36628.1), Aspergillus fumigatus narnavirus 2c (AfuNV2c; BCH36623.1), Aspergillus flavus narnavirus 1 (AfNV1; UAW09566.1), Sclerotinia sclerotiorum narnavirus 2 (SsNV2; QZE12027.1).

Supplementary Figure 7. Molecular features of Diplodia seriata botourmiavirus 1 and 2 (DsBOV1 and 2). A). A comparison of the terminal sequences of the DsBOV2 genomic RNA with that of Neofusicoccum parvum ourmia-like virus 1 (NpBOV1; MK584837.1). The viruses share high levels of nucleotide conservation. (B) Conserved RdRP motifs of DsBOV1 and 2 with other botourmiaviruses. The viruses included in the analyses are: Diplodia seriata botourmiavirus 1 (DsBOV1; OM837801), Diplodia seriata botourmiavirus 2 (DsBOV2; OM837802) Epicoccum nigrum ourmia-like virus 2 (EnOulV2; QDB75005.1), Aspergillus neoniger ourmia-like virus 1 (AnOLV1; AZT88620.1), Pyricularia oryzae ourmia-like virus 1 (PoOLV1; BBF90576.1), Phomopsis longicolla RNA virus 1 (PIRV1; YP 009345044.1), Sclerotinia sclerotiorum ourmia-like virus 1 (SsOLV1; ALD89138.1), Rhizoctonia solani ourmia-like virus 1 (RsOLV1; YP 010084710.1), Magnaporthe oryzae ourmia-like virus (MOLV; SBQ28480.1), Soybean leaf-associated ourmiavirus 2 (SLOV2; ALM62250.1).

Supplementary Figure 8. Molecular features of Diplodia seriata RNA virus 1 (DsRV1). (A) Four trans-membrane helices domains predicted at N-terminus of the ORF1 using the TMHMM - 2.0 web server. (B) Conserved motifs in RdRP domain of DsRV1 other members of the proposed family Ambiguiviridae. Four conserved motifs (IV-VIII) were observed. The viruses included in the analyses are: Diploda seriata RNA virus 1 (DsRV1; OM837806), Phoma matteucciicola RNA virus 1 (PmRV1; QNC69246.1), Periconia macrospinosa ambiguivirus 1 (PmAV1; AZT88666.1), Diaporthe ambigua RNA virus 1 (DaRV1; NP_037581.1), Magnaporthe oryzae RNA virus (MoRV; QVU39989.1), Erysiphe necator associated ambiguivirus 1 (EnAV1; QKN22641.1).

Fig. S1

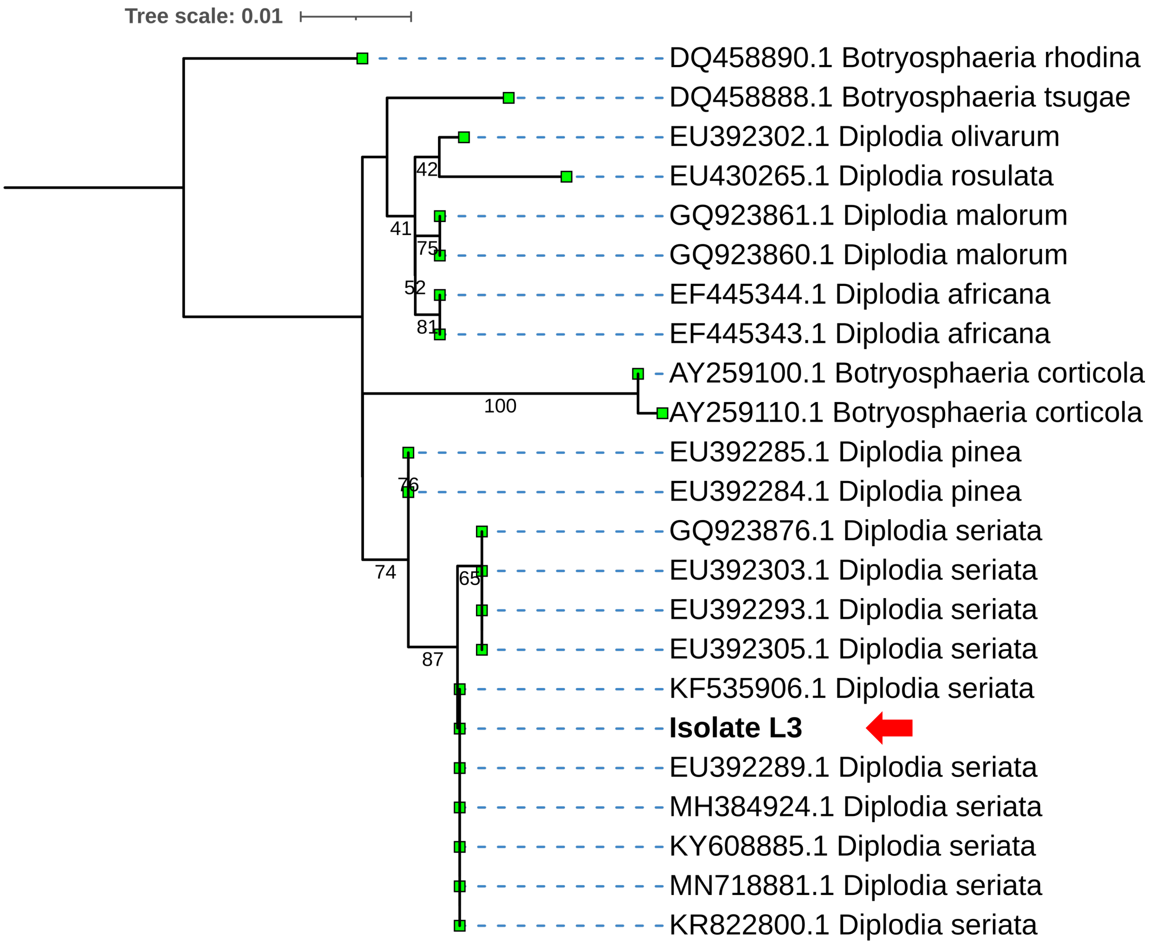

Tree scale: 0.01
DQ458890.1 Botryosphaeria rhodina
DQ458888.1 Botryosphaeria tsugae
EU392302.1 Diplodia olivarum
EU430265.1 Diplodia rosulata
GQ923861.1 Diplodia malorum
GQ923860.1 Diplodia malorum
EF445344.1 Diplodia africana
EF445343.1 Diplodia africana
AY259100.1 Botryosphaeria corticola
AY259110.1 Botryosphaeria corticola
EU392285.1 Diplodia pinea
EU392284.1 Diplodia pinea
GQ923876.1 Diplodia seriata
EU392303.1 Diplodia seriata
EU392293.1 Diplodia seriata
EU392305.1 Diplodia seriata
KF535906.1 Diplodia seriata
Isolate L3
EU392289.1 Diplodia seriata
MH384924.1 Diplodia seriata
KY608885.1 Diplodia seriata
MN718881.1 Diplodia seriata
KR822800.1 Diplodia seriata
42
41
75
52
81
100
76
74
65
87

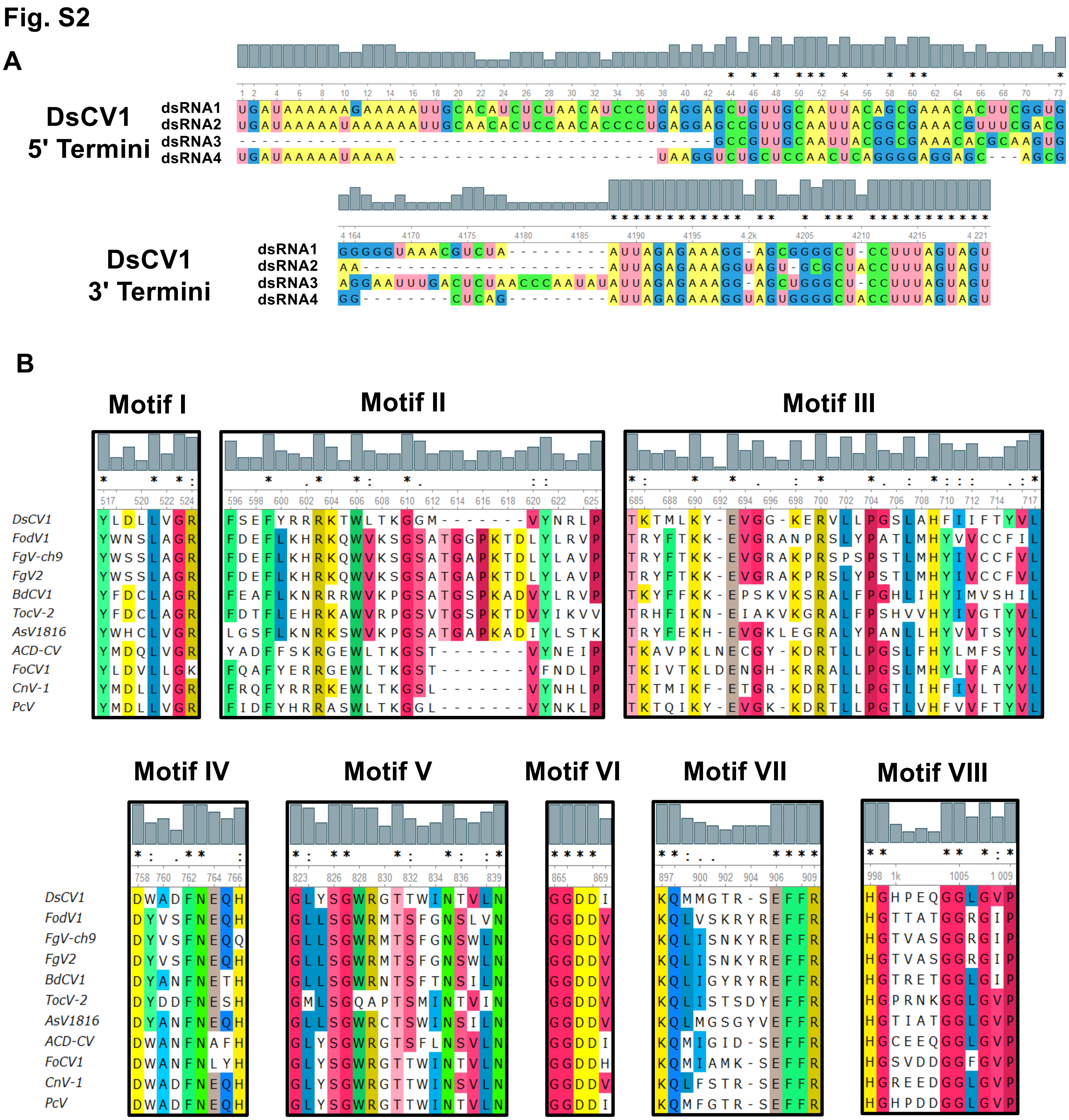
Fig. S2
A
DsCV1
5' Termini
dsRNA1
dsRNA2
dsRNA3
dsRNA4
DsCV1
3' Termini
dsRNA1
dsRNA2
dsRNA3
dsRNA4
B
Motif I
Motif II
Motif III
Motif IV
Motif V
Motif VI
Motif VII
Motif VIII
DsCV1
FodV1
FgV-ch9
FgV2
BdCV1
TocV-2
AsV1816
ACD-CV
FoCV1
CnV-1
PcV

Fig. S3

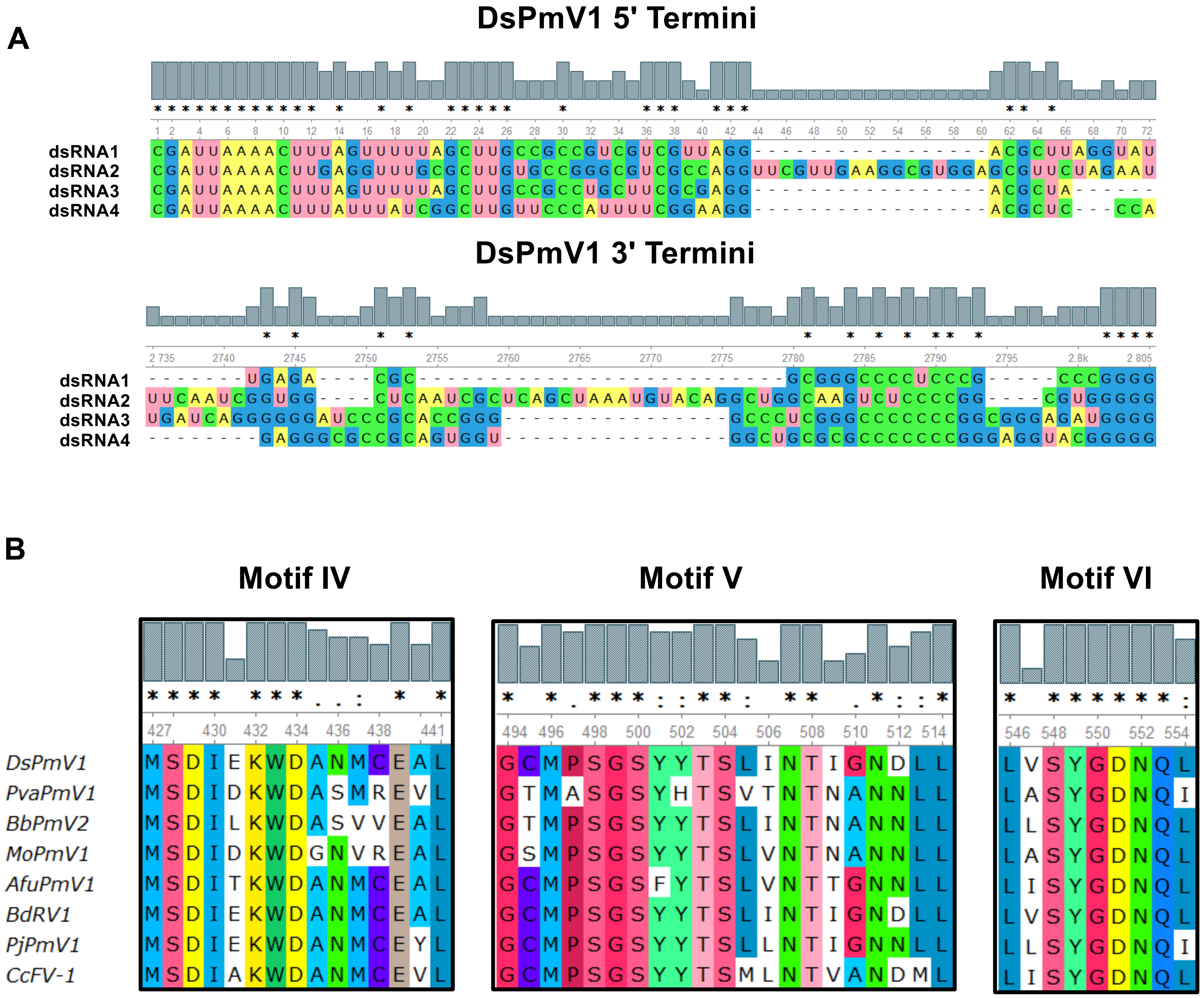

A
DsPmV1 5' Termini
dsRNA1
dsRNA2
dsRNA3
dsRNA4
DsPmV1 3' Termini
dsRNA1
dsRNA2
dsRNA3
dsRNA4
B
Motif IV
Motif V
Motif VI
DsPmV1
PvaPmV1
BbPmV2
MoPmV1
AfuPmV1
BdRV1
PjPmV1
CcFV-1

Fig. S4

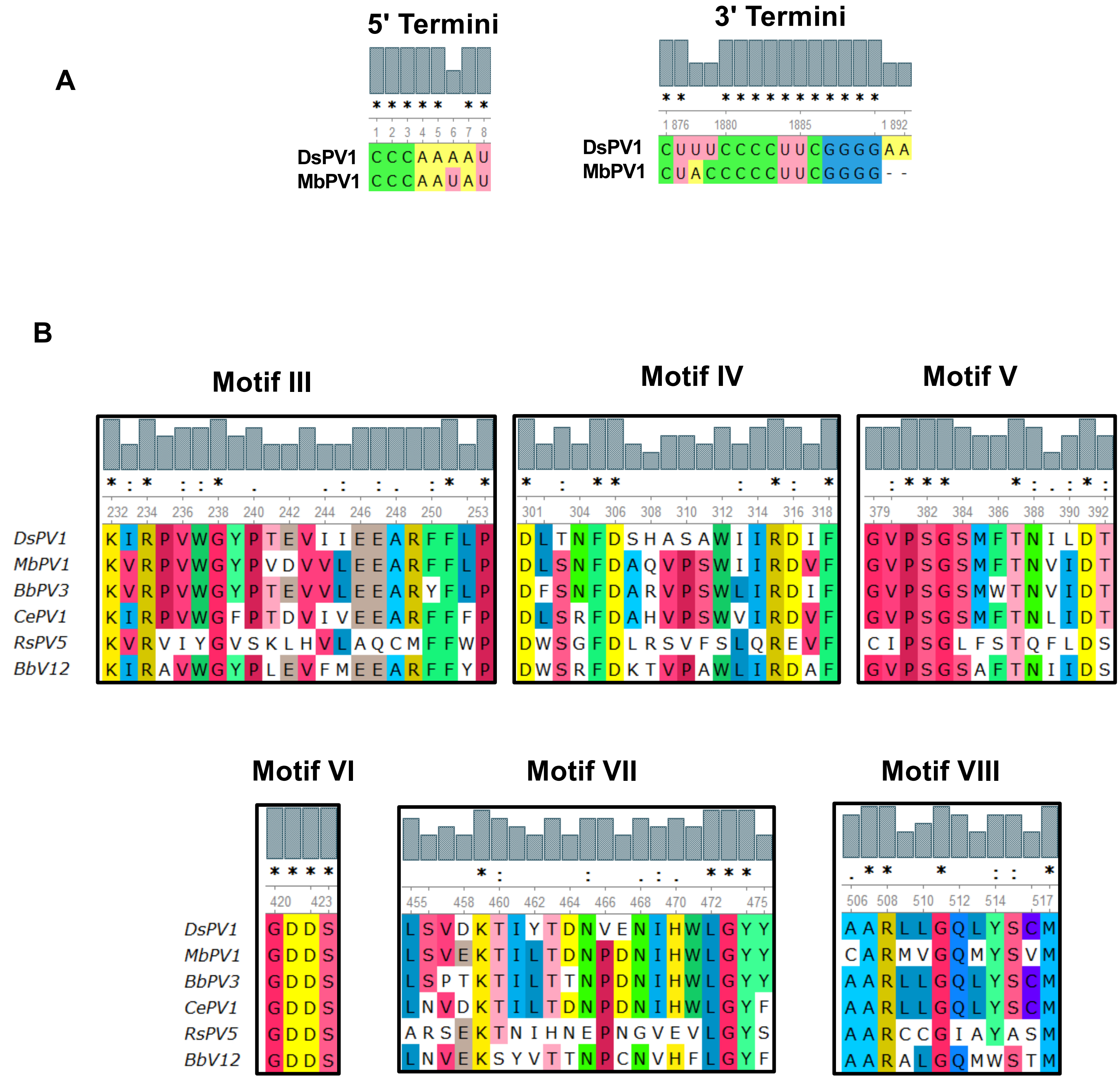
A
5' Termini
DsPV1 CCCAAAAU
MbPV1 CCCAAUAU
3' Termini
DsPV1 CUUUCCCCUUCGGGGAA
MbPV1 CUACCCCCUUCGGGG--
B
Motif III
DsPV1 KIRPVWGYPTEVIIEEARFFLP
MbPV1 KVRPVWGYPVDVVLEEARFFLP
BbPV3 KVRPVWGYPTEVVLEEARYFLP
CePV1 KIRPVWGFPTDVIVEEARFFFP
RsPV5 KVRVIYGVSKLHVLAQCMFFWP
BbV12 KIRAVWGYPLEVFMEEARFFYP
Motif IV
DsPV1 DLTNFDSHASAWIIRDIF
MbPV1 DLSNFDAQVPSWIIRDVF
BbPV3 DFSNFDARVPSWLIRDIF
CePV1 DLSRFDAHVPSWVIRDVF
RsPV5 DWSGFDLRSVFSLQREVF
BbV12 DWSRFDKTVPAWLIRDAF
Motif V
DsPV1 GVPSGSMFTNILDT
MbPV1 GVPSGSMFTNVIDT
BbPV3 GVPSGSMWTNVIDT
CePV1 GVPSGSMFTNLIDT
RsPV5 CIPSGLFSTQFLDS
BbV12 GVPSGSAFTNIIDS
Motif VI
DsPV1 GDDS
MbPV1 GDDS
BbPV3 GDDS
CePV1 GDDS
RsPV5 GDDS
BbV12 GDDS
Motif VII
DsPV1 LSVDKTIYTDNVENIHWLGYY
MbPV1 LSVEKTILTDNPDNIHWLGYY
BbPV3 LSPTKTILTTNPDNIHWLGYY
CePV1 LNVDKTILTDNPDNIHWLGYF
RsPV5 ARSEKTNIHNEPNGVEVLGYS
BbV12 LNVEKSYVTTNPCNVHFLGYF
Motif VIII
DsPV1 AARLLGQLYSCM
MbPV1 CARMVGQMYSVM
BbPV3 AARLLGQLYSCM
CePV1 AARLLGQLYSCM
RsPV5 AARCCGIAYASM
BbV12 AARALGQMWSTM

Fig. S5

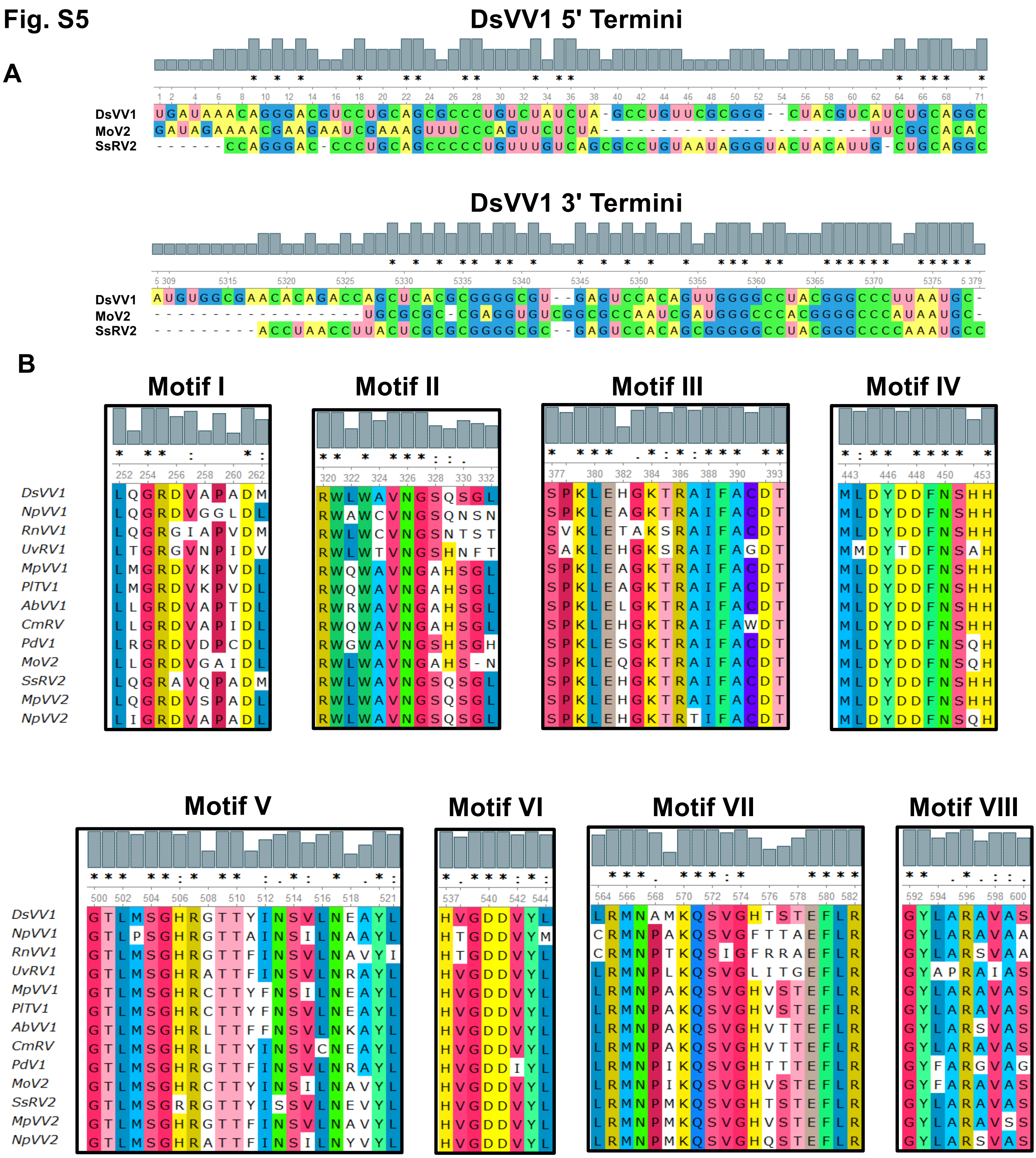
DsVV1 5' Termini
A
DsVV1
MoV2
SsRV2
DsVV1 3' Termini
DsVV1
MoV2
SsRV2
B
Motif I
Motif II
Motif III
Motif IV
Motif V
Motif VI
Motif VII
Motif VIII
DsVV1
NpVV1
RnVV1
UvRV1
MpVV1
PlTV1
AbVV1
CmRV
PdV1
MoV2
SsRV2
MpVV2
NpVV2

Fig. S6

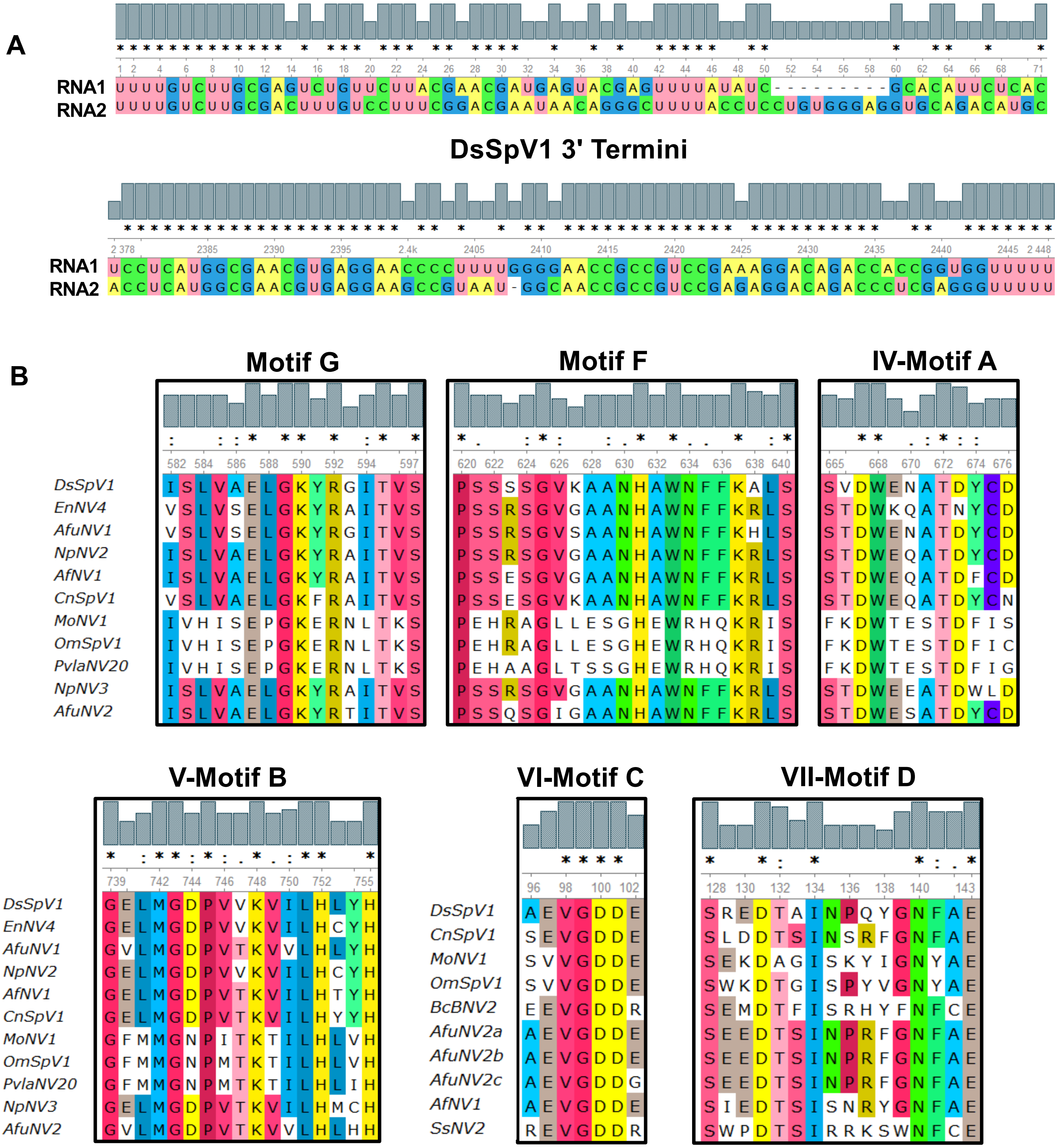

A
DsSpV1 5' Termini
RNA1
RNA2
DsSpV1 3' Termini
RNA1
RNA2
B
Motif G
Motif F
IV-Motif A
DsSpV1
EnNV4
AfuNV1
NpNV2
AfNV1
CnSpV1
MoNV1
OmSpV1
PvlaNV20
NpNV3
AfuNV2
V-Motif B
VI-Motif C
VII-Motif D
DsSpV1
CnSpV1
MoNV1
OmSpV1
BcBNV2
AfuNV2a
AfuNV2b
AfuNV2c
AfNV1
SsNV2

Fig. S7

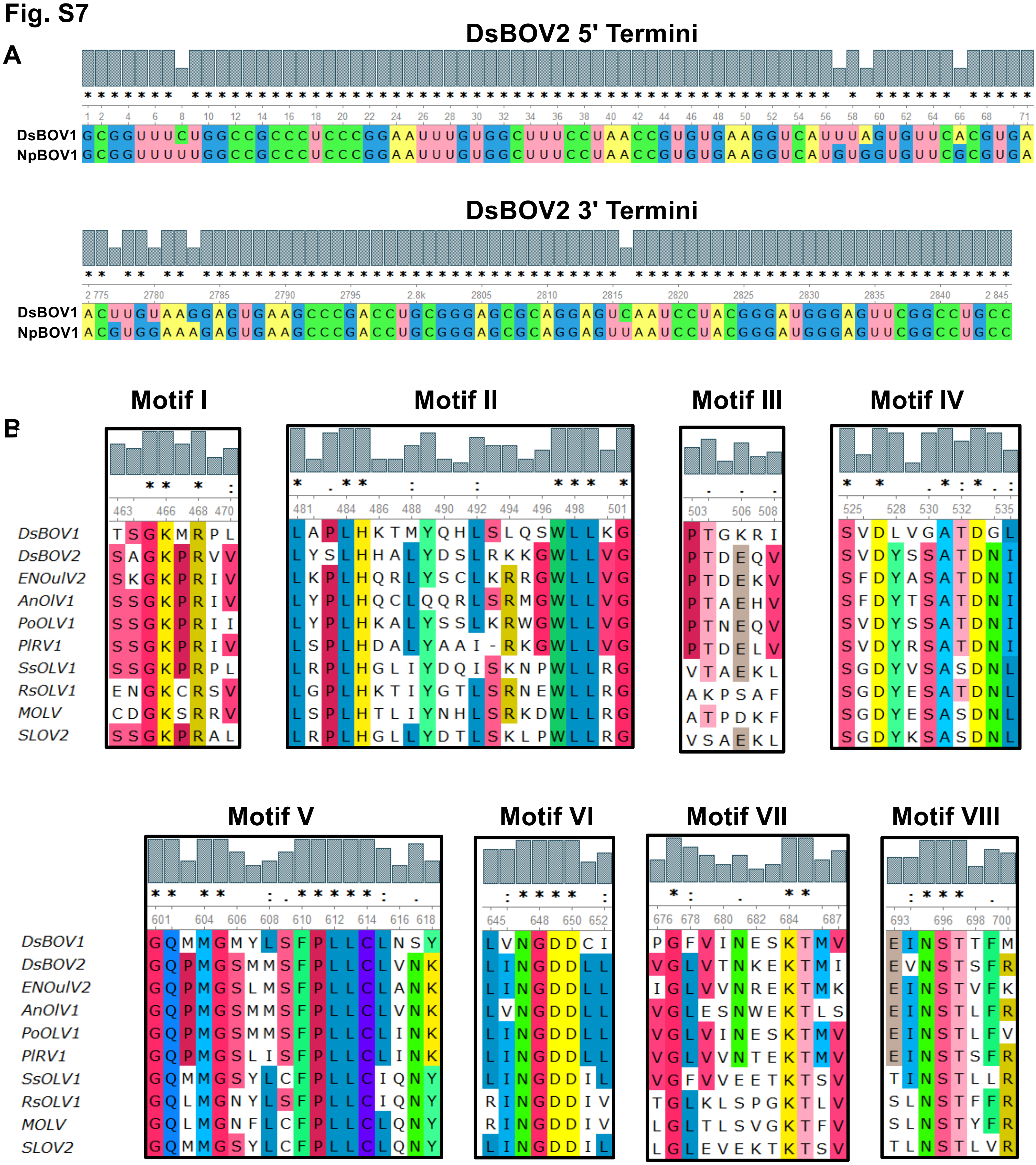
A
DsBOV2 5' Termini
DsBOV1 GCGGUUUCUGGCCGCCCUCCCGGAAUUUGUGGCUUUCCUAACCGUGUGAAGGUCAUUUAGUGUUCACGUGA
NpBOV1 GCGGUUUUUGGCCGCCCUCCCGGAAUUUGUGGCUUUCCUAACCGUGUGAAGGUCAUGUGGUGUUCGCGUGA
DsBOV2 3' Termini
DsBOV1 ACUUGUAAGGAGUGAAGCCCGACCUGCGGGAGCGCAGGAGUCAAUCCUACGGGAUGGGAGUUCGGCCUGCC
NpBOV1 ACGUGGAAAGAGUGAAGCCCGACCUGCGGGAGCGCAGGAGUUAAUCCUACGGGAUGGGAGUUCGGCCUGCC
B
Motif I
Motif II
Motif III
Motif IV
Motif V
Motif VI
Motif VII
Motif VIII
DsBOV1
DsBOV2
ENOulV2
AnOlV1
PoOLV1
PlRV1
SsOLV1
RsOLV1
MOLV
SLOV2

Fig. S8

A

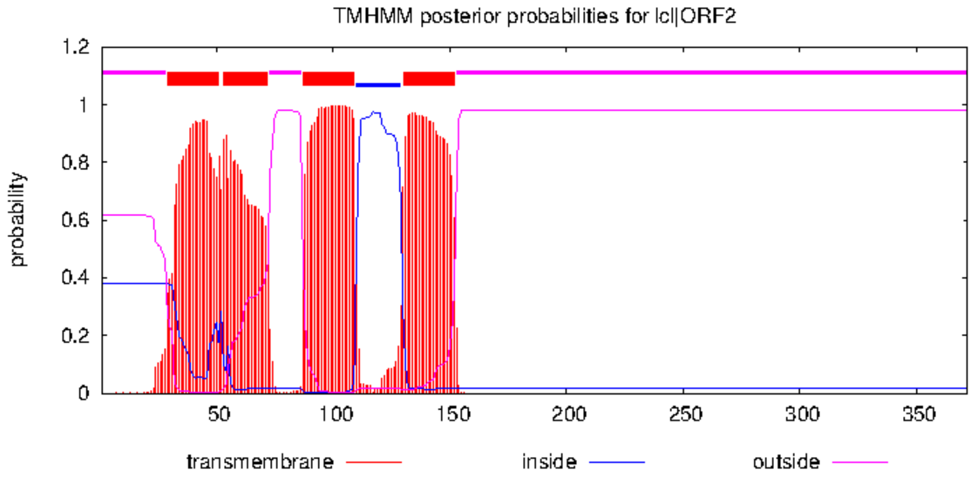
TMHMM posterior probabilities for lcl|ORF2
probability
1.2
1
0.8
0.6
0.4
0.2
0
50
100
150
200
250
300
350
transmembrane
inside
outside

B

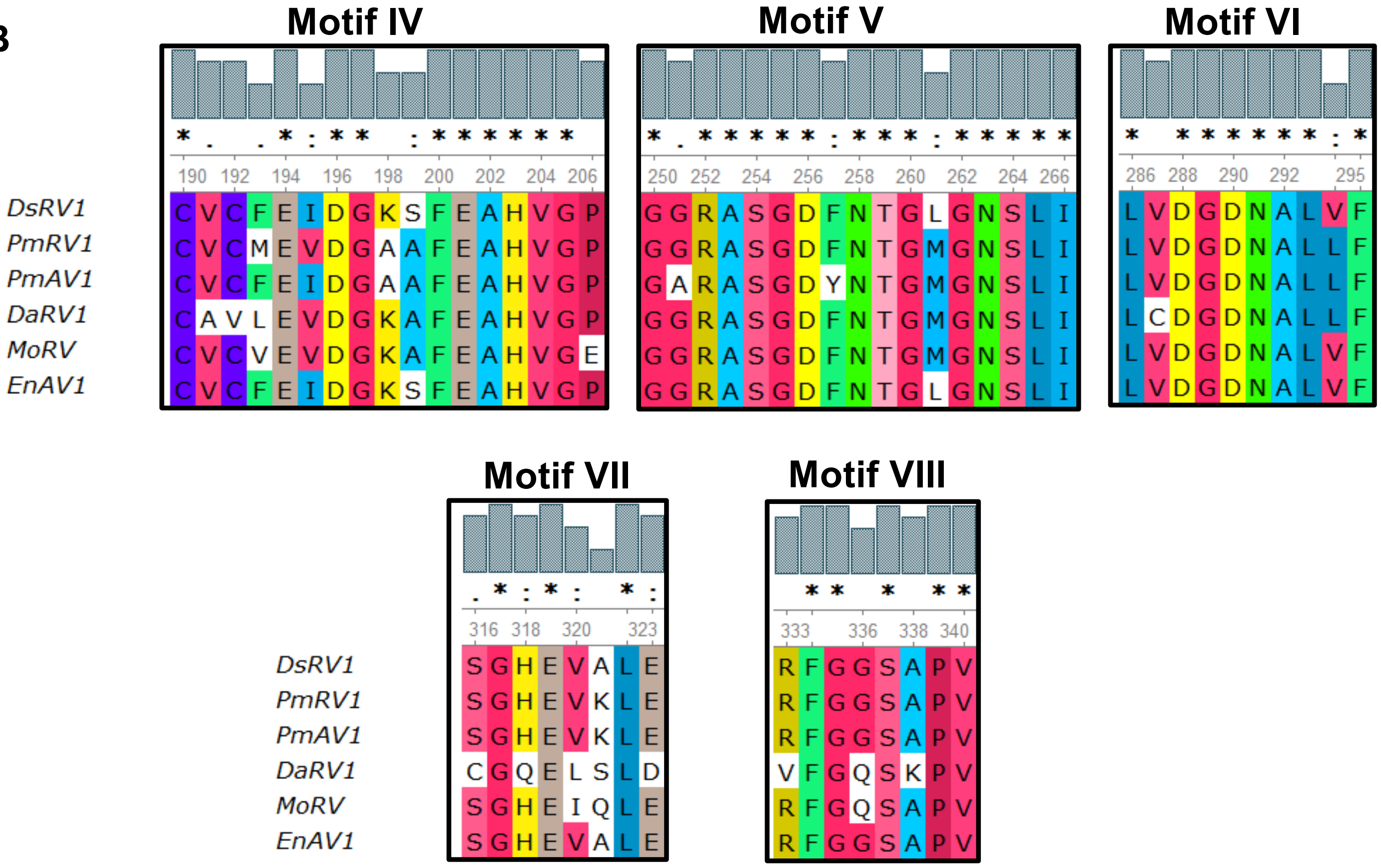
Motif IV
Motif V
Motif VI
DsRV1
PmRV1
PmAV1
DaRV1
MoRV
EnAV1
190 192 194 196 198 200 202 204 206
CVCFEIDGKSFEAHVGP
CVCMEVDGAAFEAHVGP
CVCFEIDGAAFEAHVGP
CAVLEVDGKAFEAHVGP
CVCVEVDGKAFEAHVGE
CVCFEIDGKSFEAHVGP
250 252 254 256 258 260 262 264 266
GGRASGDFNTGLGNSLI
GGRASGDFNTGMGNSLI
GARASGDYNTGMGNSLI
GGRASGDFNTGMGNSLI
GGRASGDFNTGMGNSLI
GGRASGDFNTGLGNSLI
286 288 290 292 295
LVDGDNALVF
LVDGDNALLF
LVDGDNALLF
LCDGDNALLF
LVDGDNALVF
LVDGDNALVF
Motif VII
Motif VIII
DsRV1
PmRV1
PmAV1
DaRV1
MoRV
EnAV1
316 318 320 323
SGHEVALE
SGHEVKLE
SGHEVKLE
CGQELSLD
SGHEIQLE
SGHEVALE
333 336 338 340
RFGGSAPV
RFGGSAPV
RFGGSAPV
VFGQSKPV
RFGQSAPV
RFGGSAPV

Supplementary Table S1. List of primers used in this study.

Primer name	Primer sequence (5'-3')	Amplicon size (bp)	Purpose
contig_73(F)	AAAGCGATGGTCTCCTACTTCA	552	RT-PCR
contig_73(R)	AACGTAGTCTTCAAGCTCCTGA		
contig_995 (F)	AATGCGCGGACTGTTCCGTTT	505	RT-PCR
contig_995 (R)	GATCAAGGAGGTTGGCTCGAT		
contig_218 (F)	AAGGGCACGTATTGGTTCGGAA	665	RT-PCR
contig_218 (R)	ACACCTTCGCAGATGTCGTAGT		
contig_310 (F)	GAAGTTGGCGACGATGAAGAAG	729	RT-PCR
contig_310 (R)	TTCTGCACGAACCTCGAACTC		
contig_38 (F)	AAGGTTAGGTCAGACACACACTT	493	RT-PCR
contig_38 (R)	TCCATACAAGTTGAGGCACACA		
contig_217 (F)	TCATGGTTGTTGAAGGGACCTC	770	RT-PCR
contig_217 (R)	TAGTCTGGTTCGGCCATAACAG		
contig_980 (F)	TTGCACCCGCGTTTCCTTGA	754	RT-PCR
contig_980 (R)	GCGCTACCTTGACCATGAGA		
contig_24 (F)	GCCAGTTAACTTGGTCTTCGCT	682	RT-PCR
contig_24 (R)	ACGAGGAAAGGCTCGTTGATC		
contig 33 (F)	TCATGGGGACGAACAAGTTGG	600	RT-PCR
contig 33 (R)	GGTGCTGCAACAGCCAAGTT		
Cont175 F	TTCTCTGCCATCAAATCATCGGA	519	RT-PCR
Cont175 R	TCGGAACTACTGCTTGATGCAATA		
Cont255 F	TTACATGGATCTCGTCAACACAGT	626	RT-PCR

Cont255 R	ACCAACCAAGCGACGAGTAAGT		
Cont 70 Chryso F	ACCAATGATACAGTCGCACGTCA	579	RT-PCR
Cont 70 Chryso R	AGCGCTGACAATTGGTACAGG		
Cont 348 Chryso F	GTTAGTTCGGCTCAGCGACTT	535	RT-PCR
Cont 348 Chryso R	CATCATCATGCCCATCCAGTTC		
Cont 151 Chryso F	ATCACCAGACCGTCTGTACCAT	569	RT-PCR
Cont 151 Chryso R	GTATCATCATCATCAGCTGAGGC		
Cont 965 polymy F	TGCTATCACCGTGGTGTCTCT	563	RT-PCR
Cont 965 polymy R	CTGTGTATTCCTCTCGATGCG		
Cont 245 polymy F	AGCGTAAGTACTCTGCCGTCAT	587	RT-PCR
Cont 245 polymy R	GACTCCGTGATCGACAGAATC		
Cont 706 polymy F	TCGAAATGGCCTCCGAACAAAC	533	RT-PCR
Cont 706 polymy R	CGTGAAACGGTAGGATCCATAG		
contig_980 (5P)	AACCGTTTCTTTGACATGTCCGA	397	RLM-RACE
contig_980 (3P)	TCCAGAAGCTCGGTTTCGCT	264	RLM-RACE
contig_965 (5P)	GTAGAGAGGATGTATATTGCGG	834	RLM-RACE
contig_965 (3P)	AGGATTGCCCCTCGTGTGAA	417	RLM-RACE
contig_245 (5P)	ATTGTCCTCGAGGGTTGTTAGT	561	RLM-RACE
contig_245 (3P)	GCTGCCGACGTTGAGGGTCTG	391	RLM-RACE
contig_706 (5P)	CGTACGTGGCGTGGTATTGA	357	RLM-RACE
contig_706 (3P)	GCATCACCCGCATCAAAGGAA	411	RLM-RACE
contig_24 (5P)	GGCGGTATAGTGTGTGTCCAT	714	RLM-RACE
contig_24 (3P)	TTCTACTGCGACAACGTTTTGG	654	RLM-RACE
contig 33 (5P)	CCAACTTGTTCGTCCCCATGA	300	RLM-RACE
contig 33 (3P)	TCGGCCCAAACAGCTGAAACT	500	RLM-RACE

contig_73(5P)	TAAGGACGTCCGCTTTGGTG	469	RLM-RACE
contig_73(3P)	GGAGCTTGAAGACTACGTTCG	379	RLM-RACE
contig_995 (5P)	CAACCTCGTAGAAGATCGACC	487	RLM-RACE
contig_995 (3P)	ATCCTGGGTGACGTTAAGAGAA	403	RLM-RACE
contig_218 (5P)	CCATATGTTTTCTGCATTCCAGC	448	RLM-RACE
contig_218 (3P)	AGGTATACCTGTTCTCCGTTGA	406	RLM-RACE
contig_310 (5P)	GGGTTTATCGCAGTATCTTCTC	462	RLM-RACE
contig_310 (3P)	GTTATGCCGTGAGGCAAACTT	464	RLM-RACE
contig_38 (5P)	ATCCGAAGACATACTTGCGTAAG	539	RLM-RACE
contig_38 (3P)	AAGTGAGTGGAATACGCAGTCAT	443	RLM-RACE
contig_217 (5P)	TTACACGTAAGGTTAGGGATCG	464	RLM-RACE
contig_217 (3P)	AAAGAAAAGAGAGTGGTTGGCG	512	RLM-RACE
